# Salen‐Type Copper(II) Complexes: Synthesis, Characterization, Computational Studies, Molecular Docking, Anticancer Potential, and Pharmacokinetic Prediction

**DOI:** 10.1002/open.202500061

**Published:** 2025-07-01

**Authors:** Abdellatif A. Helaly, Bandar A. Babgi, Yoji Kobayashi, Rohit K. Rai, Ehab M. M. Ali, Abdulaziz A. Kalantan, Walid M. I. Hassan, Mostafa A. Hussien, Muhammad M. I. Ismail

**Affiliations:** ^1^ Department of Chemistry Faculty of Science King Abdulaziz University (KAU) Jeddah 21589 Saudi Arabia; ^2^ Department of Chemistry Faculty of Science Damietta University Damietta 34517 Egypt; ^3^ Center of Renewable Energy and Storage Technologies (CREST) Chemistry Program Division of Physical Sciences and Engineering King Abdullah University of Science and Technology (KAUST) Thuwal 23955‐6900 Saudi Arabia; ^4^ Department of Biochemistry Faculty of Science King Abdulaziz University (KAU) Jeddah 21589 Saudi Arabia; ^5^ Department of Chemistry Faculty of Science Tanta University Tanta 31527 Egypt; ^6^ Department of Chemistry Faculty of Science Port Said University Port Said 42521 Egypt; ^7^ Department of Physics Faculty of Science Port Said University Port Said 42521 Egypt; ^8^ Department of Physics Faculty of Science Al‐Baha University Al‐Baha 65799 Saudi Arabia

**Keywords:** ADMET prediction, biological activities, copper complexes, cytotoxicity, DFT, salen‐type ligands, Schiff base

## Abstract

Transition metal complexes are considered a significant treatment for cancer diseases because of their efficacy toward cancer cells. However, most of these compounds have limited potential toward cancer cells due to their organic backbone structure. Here, the synthesis and anticancer screening of three different ligand structures of salen copper(II) complexes are reported: [Cu^II^(salophen)(H_2_O)_2_] (**1**), [Cu^II^(salen)(H_2_O)_2_] (**2**), and [Cu^II^(etho‐salen)(H_2_O)_2_] (**3**). Using density functional theory‐optimized structures, docking active site interactions are evaluated to predict the activity of salen‐type ligands and their copper(II) complexes against cyclin‐dependent kinase 5 (Cdk5 ) and aromatase cytochrome (P450) proteins. The molecular docking study reveals that among all studied ligands and complexes, [Cu^II^(salen)(H_2_O)_2_] (**2**) has the best docking score value, *S* = −8.79 and −7.73, with the lowest root mean square deviation (RMSD = 1.02 and 1.09) against proteins Cdk5 and P450, respectively. Anticancer activity against MCF‐7 and HCT‐116 cell lines reveals that [Cu^II^(salen)(H_2_O)_2_] (**2**) shows favorable behavior with IC_50_ values of 212.5 and 98.9 μm, respectively. Its parent ligand **2** shows lower potency, with IC_50_ values of 404.7 μm in MCF‐7 and 305.2 μm in HCT‐116. Notably, copper(II) complexes display reduced toxicity rather than cisplatin toward normal HFF‐1 fibroblasts, indicating a more favorable therapeutic window.

## Graphical abstract

Integrating synthesis, density functional theory studies, molecular docking, cytotoxicity, and ADMET predictions, this study identifies [Cu^II^(salen)(H_2_O)_2_] (**2**) as a selective anticancer agent. Docking and in vitro assays highlight its favorable target interactions, selective anticancer activity, and reduced toxicity toward normal fibroblasts, supporting its potential as a scaffold for safer metal‐based cancer therapies.

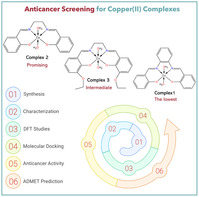

## 1 Introduction

A Schiff base is a compound that contains a carbon–nitrogen double bond (C=N), with the nitrogen atom derived from an amine and the carbon atom derived from an aldehyde or ketone.^[^ ^1^ ^]^ Schiff bases can be easily synthesized by condensation reactions between primary amines and carbonyl compounds.^[^ ^2^ ^]^ This allows for the design and synthesis of a wide range of Schiff base ligands with customized properties, such as electronic, steric, and chelating effects. These ligands can be used to tune the reactivity and selectivity of metal complexes, making them valuable in various applications.^[^ ^3^ ^]^

Salen‐type ligands and their complexes play a crucial role in the field of coordination chemistry. These ligands are derived from the condensation of salicylaldehyde and an amine, resulting in a unique structure known as a salen (N,N'‐bis(salicylidene)ethylenediamine) backbone.^[^ ^4^ ^]^ The salen backbone consists of two imine (C=N) functionalities, which serve as coordination sites for metal ions, and an ethylene bridge that imparts flexibility to the ligand. The aromatic rings derived from the salicylaldehyde moiety provide a rigid and electron‐rich platform that can be modified to fine‐tune the ligand's properties. By altering the substituents on the aromatic rings or modifying the amine component in the salen ligand, the electronic and steric characteristics can be varied, thereby influencing its coordination behavior and reactivity. The versatility and tunability of salen‐type ligands make them highly valuable in various applications, including metal complexation, asymmetric catalysis,^[^ ^5^ ^]^ and supramolecular chemistry.^[^ ^6^ ^]^

The central metal ion can vary from transition metals such as copper, iron, manganese, and nickel to main group metals. These complexes often possess unique geometries and coordination environments, influenced by the size, charge, and electronic configuration of the metal center.^[^ ^7^ ^]^ Salen copper complexes exhibit a wide range of biological applications, particularly in anticancer, antibacterial, antioxidant, and antifungal activities. In anticancer applications, some reported salen complexes were found to induce apoptosis, display selective cytotoxicity, impede the proliferation and progression of cancer cells, and inhibit tumor growth, such as in the work reported by W. Tian et al. where complexes labeled (S01), (S02), and (S03) induced cancer cell death through multiple mechanisms (mitochondrial pathway, endoplasmic reticulum (ER) stress pathway, and DNA damage pathway).^[^ ^8^ ^]^ As antibacterial agents, some reported complexes disrupt microbial membranes, inhibit vital enzymes, and induce oxidative damage.^[^ ^9^ ^]^ Additionally, salen copper complexes demonstrate antifungal effects through membrane disruption, enzyme inhibition, and reactive oxygen species (ROS) generation.^[^ ^10^ ^]^ Overall, their versatility and diverse mechanisms of action make them promising candidates for various biological applications.^[^ ^11^ ^]^

Salen‐type ligands are important in the design of metal complexes for achieving high cytotoxicity.^[^ ^12^ ^]^ They offer structural flexibility, allowing for the modification of the ligand backbone and substituents to achieve the properties of the resulting metal complex, such as lipophilicity, charge, and stereochemistry, which are crucial for optimizing the anticancer activity of the metal complexes.^[^ ^13^ ^]^ Nonsymmetric salen copper(II) complexes were used as a cofactor in the enzyme galactose oxidase (GO), the mechanism involving reduction of O_2_ to H_2_O_2_, obtaining two electrons for O_2_ reduction by the cooperative Cu^II^/Cu^I^ and tyrosyl/tyrosinate redox pairs.^[^ ^14^ ^]^ In the oxidized form of GO, Cu^II^ is coordinated to two histidines, one tyrosinate, and the tyrosinal radical. Salen ligands providing a similar N_2_O_2_ coordination environment are widely applied for the design of functional GO models and as biomimetic oxidation catalysts.^[^ ^15^ ^]^ The ability of salen copper complexes to generate ROS can induce oxidative stress in cancer cells, leading to DNA damage, protein oxidation, and ultimately cell death.^[^ ^16^, ^17^ ^]^

The above‐mentioned advantages of copper(II) as a central metal ion and salen‐type ligands and their efficacy toward cancer cells motivated us to prepare copper(II) salen and salophen complexes. These were synthesized by reactions of salicylaldehyde with ethylenediamine and phenylenediamine. Selecting these bridges controls the hydrophobicity of the resulting copper complexes, enhancing the lipophilicity and allowing the copper complexes to penetrate cell membranes, which is crucial for targeting cancer cells. The structures of all the compounds were characterized using different analytical techniques. A computational molecular docking study was performed on all salen‐type ligands, copper(II) complexes, with key residues of proteins to achieve its probable binding mode with the active sites of two receptors (PDB codes = 3eqm and 3ig7). The in vitro anticancer activities of the ligands and their copper(II) complexes were evaluated against breast MCF‐7 and colon HCT‐116 human cell lines, and normal HFF‐1 cell lines, accompanied by ADMET prediction to cover the pharmacokinetic and toxicity studies.

## 2 Experimental Section

### 2.1 Materials

All chemicals (salicylaldehyde, o‐phenylenediamine, ethylenediamine, copper acetate monohydrate, and ethanol) were purchased from Sigma‐Aldrich and used without purification.

### 2.2 Instrumentation

IR spectra were obtained on a Perkin Elmer Spectrum 100 FT‐IR spectrometer (KBr discs, 4000–400 cm^−1^). Nuclear magnetic resonance (^1^H NMR, 800 MHz) and (^13^C NMR, 100 MHz) spectra were employed on a Bruker Avance 800 MHz NMR using CDCl_3_ solvent. The powder X‐ray diffraction (PXRD) patterns were recorded on the Bruker D8 Advance X‐ray diffractometer fitted with a Cu K_α_ source of radiation and λ = 1.54056 Å. The thermal analysis of compounds was processed using a nitrogen‐atmospheric SDT 650 thermogravimetric analyzer with a rate of heating is 10 °C min^−1^ over a range of temperatures from ambient to 1000 °C. The continuous‐wave electron paramagnetic resonance (CW‐EPR) in X‐band for solid and solution samples (Bruker, Model Number: EMX Plus) was used to investigate free radicals of copper complexes at room temperature. The electrical conductivity of the compounds was measured in ethanol (10^−4^ m) solution at room temperature using an Equiptronics digital conductivity bridge (conductivity/TDS meter model Lutron YK‐22CT), where the cell constant was calibrated with 0.1 m KCl solution.^[^ ^18^ ^]^ The optical absorption spectra of ligands and complexes were measured by a PerkinElmer AA800 spectrophotometer, Model AAS UV–visible spectra with a 1.0 cm cell model.

### 2.3 Software

Perkin ChemDraw Professional 15 and Chem3D software were utilized to design all molecules. OriginPro‐2024 (Learning Edition) was utilized for graphing and analyzing the large input data.^[^ ^19^ ^]^ Using EXPO2014, the values of the crystal scheme, space group, and lattice parameters of the complexes were measured and optimized.^[^ ^20^ ^]^ The EPR spectra were interpreted by using multicomponent software^[^ ^21^ ^]^ to calculate the spectroscopic splitting factor (g‐factor). The computational studies were calculated using Gaussian 09 software through the AZIZ Supercomputer at King Abdulaziz University.

### 2.4 Molecular Docking

The molecular docking study for the synthesized salen‐type ligands and their copper(II) complexes was conducted by AutoDock4 software, gathered with AutoGrid4.^[^ ^22^ ^]^ The structures of molecules were optimized using Gaussian 09 software. The cyclin‐dependent kinase 5 (Cdk5) and aromatase cytochrome (P450) proteins (PDB ID: 3ig7 and 3eqm) were obtained from the Protein Data Bank. Receptor files were prepared using AutoDock Tools. The compounds were enclosed in a box with grid points in *x* × *y* × *z* directions (62 × 72 × 122) and a grid spacing of 0.375 Å. For each of the docking cases, the lowest energy docked conformation, according to the AutoDock scoring function, was selected as the binding mode. The 3D images were obtained by using the visualization software Discovery Studio 2020.

### 2.5 ADMET Prediction

The synthesized ligands and their copper(II) complexes were evaluated using the ADMET platform (Deep‐PK) methodology.^[^ ^23^ ^]^ Initially, the molecular structures were optimized and characterized by calculating a comprehensive set of descriptors (including lipophilicity, molecular weight, and polar surface area). These descriptors fed into the predictive models covering the major ADMET categories. The prediction workflow is mentioned in the Supporting Information.

### 2.6 Molar Ratio Method

The stoichiometric ratio of the complexes was studied using the mole ratio method.^[^ ^24^ ^]^ The metal concentration was kept constant at 10^−5^ m, while the ligand concentration was varied (0–3 × 10^−5^ m). The absorbance of sample solutions was measured in ethanol in the range of 200–800 nm at room temperature. The relation between the maximum absorbance and [M]/([L] + [M]) was plotted. The inflection of the obtained line shows the molar ratio between the ligand and metal of the formed complexes.

### 2.7 In Vitro Anticancer Activity (Supporting Information)

The in vitro cytotoxicity assay of compounds investigated on breast MCF‐7 and colon HCT‐116 human cell lines followed the literature procedures.^[^ ^25^, ^26^ ^]^ These cells were provided by the Tissue Culture Unit at the Biochemistry Department at King Abdulaziz University.

### 2.8 Synthesis of Ligands

The ligands were synthesized according to literature procedures with some modifications.^[^ ^27^, ^28^ ^]^ The ligands were synthesized by adding ethanolic solutions of the diamine to the salicylaldehyde derivatives in a 1:2 molar ratio, as shown in Scheme S1, Supporting Information. The mixture was refluxed with stirring for 3 h, and the products were collected after filtration, washing, and drying in the oven at 60 °C.

Ligand (**1**): It was prepared by adding an ethanolic solution of o‐phenylenediamine (3 mmol) to salicylaldehyde (6 mmol) to obtain a yellowish‐brown powder. **M.P.** = 162 °C, **IR** (KBr) ν (cm^−1^): 1620 (C=N), 1282 (C—O). ^**1**^ **H NMR** (CDCl_3_, *δ*/ppm): 6.7(m, Ar‐H), 6.82(m, Ar‐H), 7.01(m, Ar‐H), 7.18(m, Ar‐H), 8.43(s, HC=N), 10.79(s, OH). ^**13**^ **C NMR** (CDCl_3_, *δ*/ppm): 117.41, 119.02, 119.27, 120.09, 127.61, 132.40, 133.35, 142.58, 161.24, and 163.79.

Ligand (**2**): It was synthesized by dissolving ethylenediamine (3 mmol) in hot ethanol and then adding salicylaldehyde (6 mmol) to the reaction solution to obtain a yellow precipitate. **M.P.** = 125 °C, **IR** (KBr) ν (cm^−1^): 1639 (C=N), 1288 (C—O). ^**1**^ **H NMR** (CDCl_3_, *δ*/ppm): 3.93(s, 4H, bridge‐CH_2_), 6.82(m, Ar‐H), 6.96(m, Ar‐H), 7.21(m, Ar‐H), 7.26(m, Ar‐H), 8.34(s, HC=N). ^**13**^ **C NMR** (CDCl_3_, δ/ppm): 59.64, 116.89, 118.92, 131.62, 132.72, 161.07, and 166.59.

Ligand (**3**): It was prepared by dissolving ethylenediamine (3 mmol) in hot ethanol. Additionally, ethoxy salicylaldehyde (6 mmol) was added to form a yellow precipitate. **M.P.** = 140 °C, **IR** (KBr) ν (cm^−1^): 1637 (C=N), 1260 (C—O). ^**1**^ **H NMR** (CDCl_3_, *δ*/ppm): 1.46(S, CH_3_), 3.96(S, bridge‐CH_2_), 4.12(S, O—CH_2_), 6.78(m, Ar‐H), 6.84(m, Ar‐H), 6.91(m, Ar‐H), 7.29(m, Ar‐H), 8.34(S, HC=N), 13.63(S, OH). ^**13**^ **C NMR** (CDCl_3_, *δ*/ppm): 14.91, 59.46, 64.66, 115.28, 118.05, 118.92, 123.08, 147.69, 151.51, and 166.59.

### 2.9 Synthesis of Complexes

**Scheme 1:**
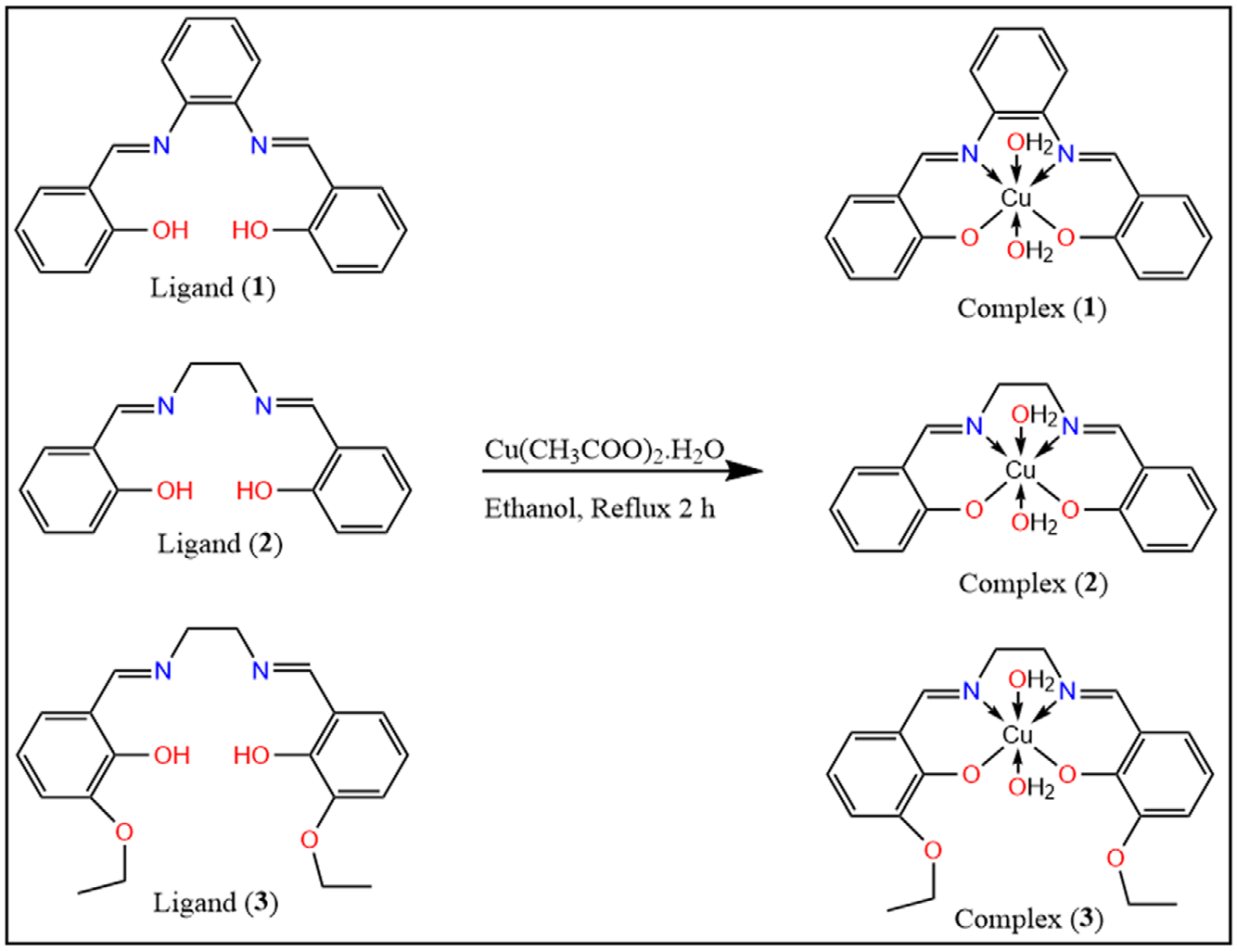
Synthesis of copper salen and salophen complexes.

Complex (**1**): As shown in **Scheme** **1**, it was synthesized by adding (1 mmol) of ligand **1** to (1 mmol) of copper(II) acetate monohydrate (CH_3_COO)_2_Cu.H_2_O, then refluxed for 2 h with stirring. The brown ppt was separated by filtration, washing, and drying in an oven. **M.P.** >300 °C, **IR** (KBr) ν (cm^−1^): 1611 (C=N), 619 (Cu—O), 501 (Cu—N), and 1338 (C—O).

Complex (**2**): As shown in Scheme 1, it was synthesized by adding (1 mmol) of ligand **2** to (1 mmol) of copper(II) acetate monohydrate (CH_3_COO)_2_Cu.H_2_O, then refluxed for 2 h with stirring. The green ppt is separated by filtration, washing, and drying in an oven. **M.P.** >300 °C, **IR** (KBr) ν (cm^−1^): 1629 (C=N), 618 (Cu—O), 471 (Cu—N), and 1354 (C—O).

Complex (**3**): As shown in Scheme 1, it was synthesized by adding (1 mmol) of ligand **3** to (1 mmol) of copper(II) acetate monohydrate (CH_3_COO)_2_Cu.H_2_O, then refluxed for 2 h with stirring. The black ppt was separated by filtration, washing, and drying in an oven. **M.P.** >300 °C, **IR** (KBr) ν (cm^−1^): 1623 (C=N), 610 (Cu—O), 463 (Cu—N), and 1312 (C—O).

## 3 Result and Discussion

The results of the physicochemical analyses gathered with the density functional theory (DFT) calculations of the synthesized salen‐type ligands (**1–3**) and their copper(II) complexes (**1–3**) demonstrate the nature of the chemistry between the ligands and copper metal. The stoichiometric ratio for the reaction between copper ions and salen‐type ligands was obtained to be 1:1 metal‐ligand. The air resistance and the solubility test indicate its solubility in common organic solvents, as well as its stability in air. The geometry structure of chelates is illustrated to be octahedral through ONNO salen ligands and two water molecules, which is also validated by DFT calculations. The molar conductivity of the obtained salen ligands and their complexes was carried out in ethanol to show the nonelectrolytic nature of these compounds.

The optical absorption bandgaps, which were obtained experimentally and computationally, showed that the ligands (**1–3**) and complexes (**1–3**) are insulator materials, except complex **1** can be considered as semiconductor materials with a value around 2.67 eV. The thermal stability of the compounds showed that the complexes are more stable than their ligands. The several diffraction peaks in the PXRD charts confirmed the polycrystalline nature of the prepared complexes. The molecular docking studies illustrated that complex **2** has the best activity toward the receptors, both 3eqm and 3ig7, among all other compounds with the highest binding energy score (most negative) and low RMSD. The biological anticancer activity showed that among all compounds, complex **2** is considered the best anticancer agent toward MCF‐7 and HCT‐116 cell lines, with a higher selectivity index than cisplatin reference.

### 3.1 Molar Ratio Method

**Figure 1:**
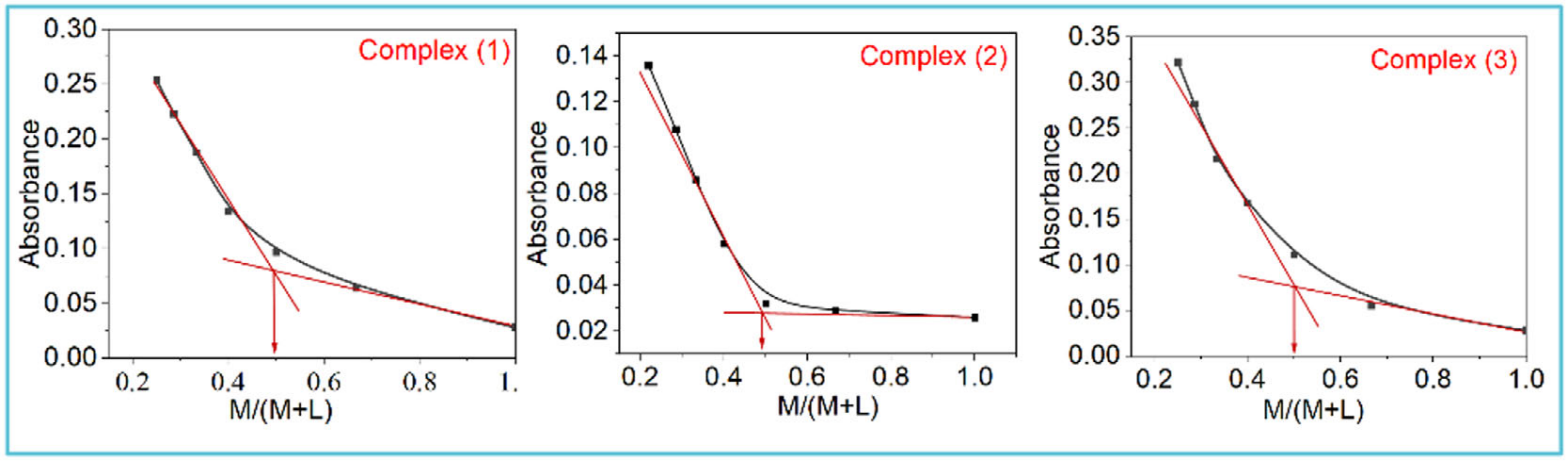
Molar ratio graphs of copper(II) complexes (**1**), (**2**), and (**3**).

The molar ratio method was employed to examine the complexes’ stoichiometry.^[^ ^29^ ^]^ The absorbance values of each addition of metal ion to ligand, as shown in Table S1, Supporting Information, were plotted against the ratio [*M*]/([*L*] + [*M*]) and presented in **Figure** **1**. According to the results, a ratio of 1:1 metal to ligand was observed in all complexes at an inflection point of 0.5.

### 3.2 Conductivity and Infrared Spectra

The molar conductivity of the ligand and its metal complexes was investigated in ethanol at (1×10^−4^ m), and it was in the range of 0.17–1.8 μS. The low conductance values of compounds confirm their nonelectrolytic nature.^[^ ^30^ ^]^

The infrared (IR) spectra of the Schiff bases showed absorption bands around 1620 cm^−1^ attributed to the C=N stretching mode, as illustrated in Figure S1, Supporting Information. The important information can be obtained by comparing the IR peak of ligands with the Cu(II)‐ligand complex. The C=N stretching mode of the free ligands (**1–3**) occurs at 1620, 1639, and 1637 cm^−1^, respectively, which have been shifted to 1611, 1629, and 1623 cm^−1^ by the formation of copper(II) salen complexes (**1–3**), respectively.^[^ ^31^, ^32^ ^]^ The C—O stretching mode for ligands (**1**‐**3**) occurs at 1282, 1288, and 1260 cm^−1^, respectively, which have been shifted to 1338, 1354, and 1312 cm^−1^ by forming copper(II) complexes, respectively. The appearance of Cu–O and Cu–N peaks for complexes and disappearance in ligands, as shown in Table S2, Supporting Information, suggesting the coordination of N and O with Cu(II) ion.

### 3.3 Electronic Spectra

Figure S2a,b, Supporting Information, shows the optical absorption spectra of synthesized ligands and their complexes (200–500 nm). For each ligand, Figure S2a, Supporting Information shows more than one absorption peak, which confirms the presence of many electronic transitions for them. Figure S2b, Supporting Information, illustrates d–d transitions (500–700 nm, very broad but weak due to selection rules), metal‐ligand charge transfer (MLCT) or related (high absorption, below 400 nm), and *π*–π* from the phenyl group (strong, below 500 nm) are the relevant transitions. Figure S3 and S4, Supporting Information, show the energy gap values of the ligands (**1–3**) and complexes (**1–3**). The experimental and computational optical absorption spectra are matched as shown in Table S3, Supporting Information.

### 3.4 EPR and NMR Spectra

The NMR spectra were recorded in CDCl_3_ solution, and the spectral data are presented in experimental Section 2.8. As shown in Figure S5–S10, Supporting Information, the NMR spectra illustrate the formation of imine bond C=NH for all ligands (**1–3**); all details are mentioned in Section S3.4, Supporting Information.

EPR spectra were interpreted using Multicomponent software,^[^ ^21^ ^]^ as shown in Table S4 and Figure S11, Supporting Information, to calculate the spectroscopic splitting factor (g‐factor) due to the Zeeman effect.^[^ ^33^ ^]^ The g‐factor for complexes **1, 2,** and **3** has a small difference between g_||_ and g_⊥_; therefore, the g‐factor is nearly axially symmetric. As can be shown, for complex **1**, g_||_ = g_⊥_, indicative of normal octahedral symmetry for coordinated ligands, whereas for complex **2,** g_||_ > g_⊥_, indicative of slightly elongated octahedral symmetry for coordinated ligands, and for complex **3,** g_||_ < g_⊥_, indicative of slightly compressed octahedral symmetry for the coordinated ligands.^[^ ^34^ ^]^

The *G* value is used to estimate the strength of the exchange interaction between paramagnetic ions in solid materials that can be calculated utilizing the formula *G* = (g_||_ −2.002)/(g_⊥_ −2.002).^[^ ^35^ ^]^ The G value is 1, 5.75, and −1.25 for complexes **1, 2,** and **3**, respectively, indicative of a relatively weak interaction between copper ions. Additionally, the *G* value is used to assess the degree of deviation of the g‐factors from the free electron value of 2.002. The *G* value close to 0 indicates an isotropic g‐factor, where the g_||_ and g_⊥_ components are similar, as in the case of complex **1**. A positive *G* value suggests a g‐factor that is larger in the parallel direction, as in the case of complex **2**, while a negative *G* value indicates a g‐factor that is larger in the perpendicular direction, as in complex **3**. The present EPR results show that g_||_ is lower than the 2.3 value, suggesting that the covalent character of metal–ligand bonds in the copper(II) complexes.^[^ ^35^ ^]^ Additionally, the effective magnetic moments of copper complexes were determined to be 1.73 BM for complexes **1** and **3** and 1.74 BM for complex **2**, as shown in Table S4, Supporting Information, by utilizing the equation *μ* ^2^_eff_ = ¾ (*g* ^2^_av_), indicating the presence of one electron in the copper ion.

### 3.5 Thermal Gravimetric Analysis (TGA)

**Figure 2:**
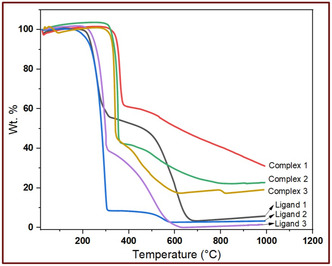
TGA graphs of ligands and their complexes.

**Figure** **2** and Table S5, Supporting Information, show the thermal decomposition of the salen‐type ligands and their complexes, processes started around 200 °C and ended at 1000 °C, suggesting that the water present in Cu(II) complexes would be coordinated water.^[^ ^36^ ^]^ The discussion of ligands **(1–3)** is illustrated in Section S3.5, Supporting Information.

Complex **1** decomposed in two stages: the first stage occurs at 306–406 °C due to the loss of C_6_H_16_N_2_O_3_ (found 39.58%, calc. 39.67%); the second stage occurs at 406–1000 °C due to the loss of C_10_H_2_ (found 29.23%, calc. 29.51%), and it has C_4_ + CuO as residue over 1000 °C (found 31.19%, calc. 30.82%). Complex **2** has two decomposition stages: the first stage takes place at 304–386 °C due to C_10_H_18_N_2_O_3_ (found 58.33%, calc. 58.56%); and the second stage occurs from 386 to 800 °C due to C_6_ (found 19.69%, calc. 19.7%), and the copper(II) oxide was residue over 800 °C. Complex **3** has lost 57.12 wt% at 283–376 °C because of C_11_H_19_N_2_O_5_ and 25.75 wt% at 376–639 °C due to C_9_H_7_, and over 639 °C, the copper(II) oxide was the residue. This decomposition consists of the elimination of the coordinated water, followed by the degradation of the organic ligand, and then the leftover carbon atoms and metal oxide.^[^ ^37^ ^]^

### 3.6 PXRD

Figure S12 and Table S6, Supporting Information show the X‐ray diffraction pattern of the prepared complexes, as described in Section S3.6, Supporting Information. The observed diffraction peaks confirm the polycrystalline nature of the prepared complexes.

### 3.7 Computational Studies

**Figure 3:**
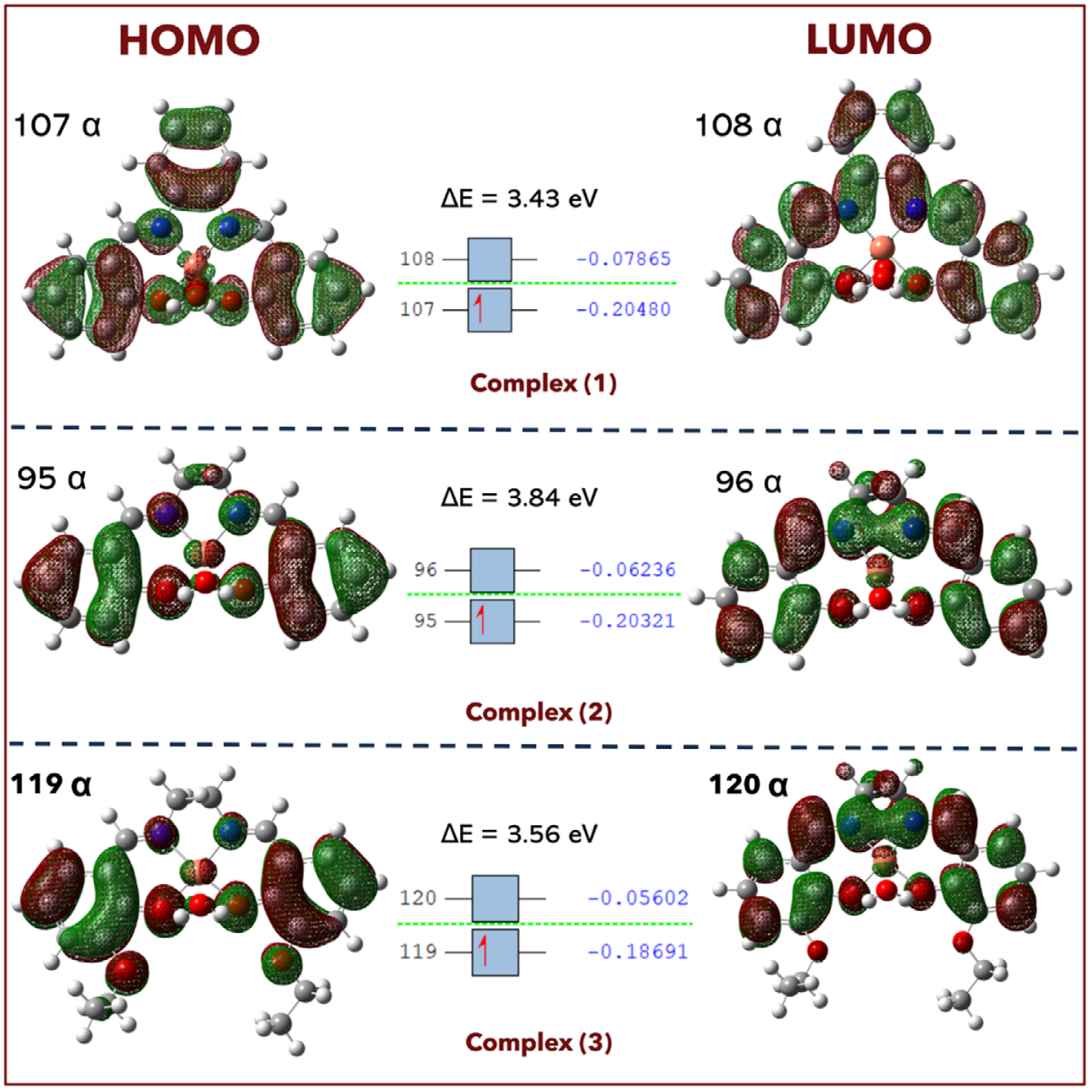
Molecular orbitals (HOMO) and (LUMO) of the complexes (**1–3**).

Gaussian 09, Revision A.02, was used to perform density functional theory (DFT) studies.^[^ ^38^ ^]^ The highest occupied molecular orbitals (HOMO) and lowest unoccupied molecular orbitals (LUMO) of the ligands and their complexes (**Figure** **3** and S13, Supporting Information) are helpful parameters that are recognized as the frontier molecular orbitals.^[^ ^39^ ^]^ Their energies (*E*_HOMO_, *E*_LUMO_) are all negative, demonstrating the products’ stability as illustrated in **Table** **1**. The energy gap (Δ*E* = *E*_LUMO_ − *E*_HOMO_), which is in place inside the LUMO‐HOMO molecule, characterizes the charge transfer interaction.^[^ ^40^ ^]^

**Table 1:** The quantum parameters calculated for ligands and their complexes.

Compd.	*E*_HOMO_ [eV]	*E*_LUMO_ [eV]	Δ*E* [eV]	*χ* [eV]	*η* [eV]	*σ* [eV^−1^]	Pi [eV]	S [eV^−1^]	ω [eV]	Δ*N*_max_ [eV]
L1	−5.61	1.44	4.16	3.52	2.08	0.48	−3.52	0.24	2.98	1.69
L2	−5.69	−1.22	4.46	3.46	2.23	0.45	−3.46	0.22	2.68	1.55
L3	−5.28	−1.20	4.08	3.24	2.04	0.49	−3.24	0.24	2.57	1.59
C1	−5.58	−2.15	3.43	3.86	1.71	0.58	−3.86	0.29	4.35	2.25
C2	−5.52	−1.69	3.84	3.61	1.92	0.52	−3.61	0.26	3.39	1.88
C3	−5.09	−1.52	3.56	3.31	1.78	0.56	−3.31	0.28	3.07	1.85

Table 1 shows the absolute hardness (*η*), absolute softness (*ω*), global softness (*σ*), chemical potentials (Pi), global electrophilicity (*S*), absolute electronegativity (*χ*), and added electronic charge (Δ*N*_max_), which have been estimated using Equation. $\chi \;=\;-\left(E_{\text{LUMO}}+E_{\text{HOMO}}\right)/2$, $\eta \;=\;\left(E_{\text{LUMO}}-E_{\text{HOMO}}\right)/2$, σ =1/η, Pi= –χ, S= 1/2η, $\omega ={\text{ Pi}}^{2}/2\eta$ and $\Delta N_{\text{max}}\;=\;-\text{Pi}/\eta$.^[^ ^41^ ^]^

Figure S14, Supporting Information, illustrates the molecular structure, numbering of atoms in the ligands, and their complexes. By emphasizing the most significant bond lengths and bond angles for compounds as demonstrated in Table S7, Supporting Information, conclusions can be drawn, include I) in complexes (**1–3**), deprotonation occurs at O(17) and O(18) to form the Cu(II)—O bond. II) The significant change of bond length of N(4)–C(2), N(3)–C(1), O(18)–C(12), and O(17)–C(11) through the complexation and the shift of bond angles O(18)–C(12)–C(6), O(17)–C(11)–C(5), N(4)–C(2)–C(1), and N(3)–C(1)–C(2) in all complexes (**1–3**) is an evidence of metal bond formation.^[^ ^42^ ^]^ III) The energy values of *E*_HOMO_ are an indication of the strength of electron‐donor molecules; for instance, the higher *E*_HOMO_ values suggest a strong electron‐donor molecule and the inverse. *E*_LUMO_ values refer to the potential of electron‐acceptor molecules.^[^ ^43^ ^]^

Compared to rigid molecules, soft molecules have smaller energy gaps, which makes them more reactive (as opposed to the high energy gaps of hard molecules) and can readily donate electrons toward an acceptor. In a coordination system, the metal ion acts as a Lewis acid, while the ligand acts as a Lewis base. As a result, it has been discovered that ligands with the proper value (*σ*) have a high potential for successfully interacting with ions of metal;^[^ ^44^ ^]^ this is also supported by the ligand's predicted chemical potential (Pi). The reactivity coefficient predicts the relative stability of the system regarding electron transfer reactions; besides the electrophilicity index (*χ*), it is a measure of the ability of a system to act as an electrophile species that can accept a pair of electrons from a nucleophile. The ligand binds to copper(II) via O(17) and O(18) of the salicylaldehyde moiety as well as N(3) and N(4) of the Schiff base group. These atoms have a higher electronegative charge, as shown in Table S8, Supporting Information according to the natural bond orbital (NBO) analysis, which support the existence of robust coordination environments.

According to the computational studies using time‐dependent density functional theory (TD‐DFT), Figure S15, Supporting Information, Complex **1** has transitions from 105*α* → 108*α* and 106*β* → 107*β*, which leads to a peak at 2.8 eV with *f* = 0.023. Complex **2** has transitioned from 93*α* → 97*α* and 94*β* → 95*β*, which leads to the appearance of a peak at 2.8 eV with *f* = 0.013. Complex **3** appeared to peak at 2.99 eV with *f* = 0.003 because of the electronic transition from orbital 119*α* → 120*α* and 118*β* → 119*β*. These bands are mainly due to metal‐to‐ligand charge transfer (MLCT) in combination with ligand‐to‐ligand charge transfer (LLCT).

### 3.8 Molecular Docking

**Figure 4:**
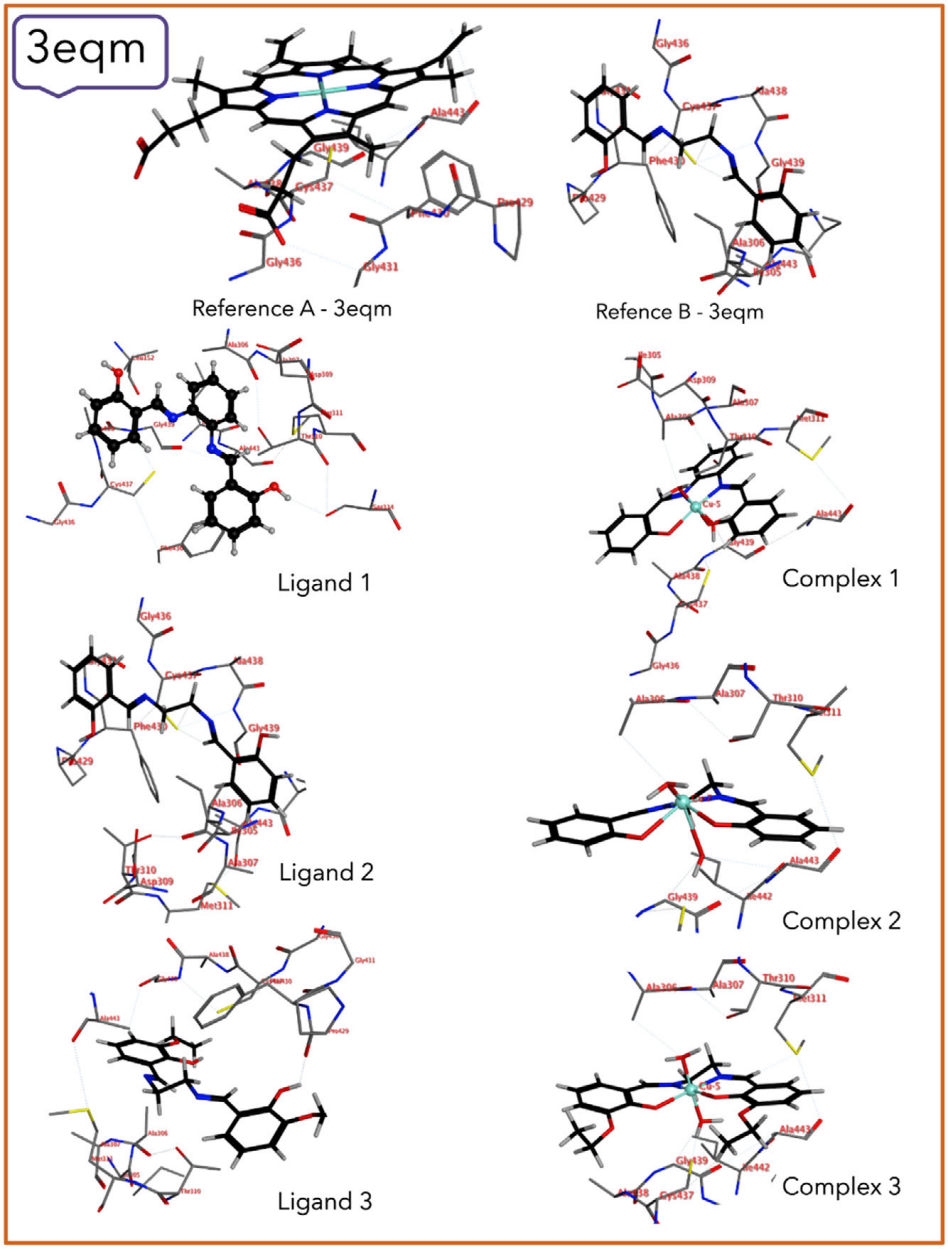
Ligand and complex interactions with the 3eqm‐receptor.

**Figure 5:**
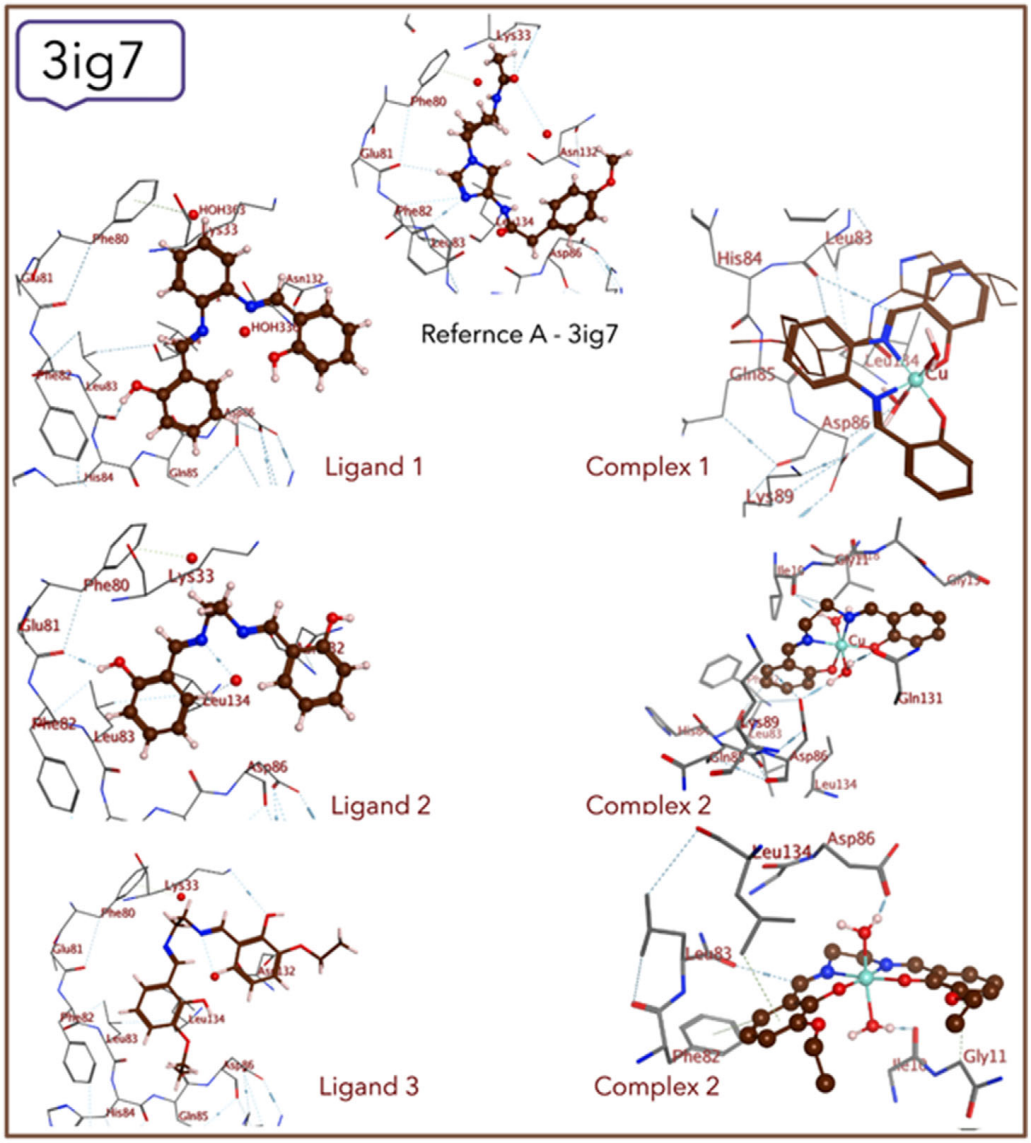
Ligand and complex interactions with the 3ig7‐receptor.

Because of the significance of theoretical studies of protein–ligand interactions,^[^ ^45^ ^]^ the molecular docking of the salen‐type ligands (**1–3**) and their complexes (**1–3**) against various proteins with two specific receptors (PDB ID: 3eqm and 3ig7) was investigated using the AutoDock4 software.^[^ ^46^ ^]^ The 3D graphs of protein–ligand interactions for compounds against the receptor are presented in **Figure** **4** and **5**.

Molecular docking is a helpful technique to give an indication of the stable interactions between proteins and synthesized compounds. The activity of ligands (**1–3**) and their complexes (**1–3**) is determined by the binding affinity between them and receptors.^[^ ^47^ ^]^ The value of the minimal binding energy score for compounds illustrated that the complexes have the highest binding compared to their parent ligands with the lowest scoring energy, as shown in **Table** **2**.^[^ ^48^ ^]^ The low binding energy value and low RMSD for complexes with each receptor give great evidence for stability and good docking behavior, more than the free ligand.^[^ ^49^ ^]^ Finally, complex **2** presented the best activity toward both receptors 3eqm and 3ig7 among all other compounds, with the highest binding energy score and low root mean square docking. This also matches with the experimental anticancer activities against breast MCF‐7 and colon HCT‐116 cancer cell lines.^[^ ^50^ ^]^

**Table 2:** Docking score of compounds with receptors (3eqm and 3ig7).

	3eqma)		3ig7b)	
Comp.	S	rmsd‐Refine	S	rmsd‐Refine
Reference A – 3eqm	−14.1	1.51	–	–
Reference B – 3eqm	−7.60	1.09	–	–
Reference A – 3ig7	–	–	−7.35	1.63
Ligand 1	−6.17	1.34	−6.44	1.65
Ligand 2	−8.65	1.07	−7.14	1.28
Ligand 3	−7.17	1.19	−6.64	1.46
Complex 1	−6.56	1.11	−6.61	1.55
Complex 2	−8.79	1.02	−7.73	1.09
Complex 3	−7.55	1.04	−6.65	1.16

a) Reference A‐3eqm and Reference B‐3eqm are the native ligands of 3eqm receptor.

b) Reference A‐3ig7 is the native ligand of 3ig7 receptor.

Analyzing the amino acid interactions with the ligands and their copper(II) complexes has given important insights into how these compounds interact with the P450 protein (PDB = 3eqm) through molecular docking analysis. To compare the synthesized compounds with the reference compounds (native protein ligand), references A and B are used. Reference A‐3eqm mostly interacts with Gly 431, whereas reference B‐3eqm interacts with Cys 437 as well as Gly 431. Among the synthesized ligands, ligand **1** interacts with both Ser 314 and Ala 437, whereas ligand **2** only interacts with Cys 437. Ligand **3** interacts with Pro 429. Among the complexes, complex **1** exhibits interaction with multiple amino acids, such as Ala 306, Gly 439, and Cys 437. Complex **2** interacts with Ala 306, Gly 439, and Ala 443, whereas complex **3** interacts with Ala 306, Gly 439, Met 311, and Cys 437, highlighting the diversity in binding patterns and indicating that the complexation enhances the docking of complexes rather than their parent ligands.

For the molecular docking results against the CDK‐5 (PDB = 3ig7) protein, reference A showcases connections with Leu 83, Glu 81, and Lys 33, indicating a specific binding profile. In contrast, ligand **1** interacts with His 84, ligand **2** with Lys 89 and Leu 298, and ligand **3** with Glu 8, delineating a diverse range of binding patterns across the compounds. Among the complexes, complex **1**, **2**, and **3** display interactions with Asp 86 and Ile 10, with additional associations to Leu 83 in complex **1** and complex **3** and Gln 131 in complex **2**. Noteworthy is the resemblance of complex **3** to reference A‐3ig7, sharing interactions with Leu 83 and Asp 86, suggesting potential binding mode similarities. However, the absence of interactions with Glu 81 and Lys 33 in complex **3** indicates distinct binding interactions compared to reference A‐3ig7.

### 3.9 ADMET Prediction

The in silico ADMET analysis presented clear pharmacokinetic profiles of free salen‐type ligands and their copper(II) complexes. Absorption, free ligands showed high intestinal absorption and were predicted to be orally bioavailable, while metal complexes exhibited lower bioavailability probabilities, as shown in Table S9, Supporting Information. Distribution, as illustrated in Table S10, Supporting Information, the predictions indicate that copper(II) complexes were classified as penetrable with high confidence, indicating a strong potential to access the central nervous system. In contrast, the free ligands either lacked blood brain barrier (BBB) permeability or had low‐confidence predictions, with ligand 1 being the only one marked as penetrable (0.553). This suggests that metal complexation may enhance the ability of these compounds to cross the BBB. Metabolism, the predictions showed that none of the ligands and copper(II) complexes were predicted to inhibit the CYP3A4 enzyme, as shown in Table S11, Supporting Information. All ligands were identified as potential substrates of CYP2D6, though the predictions carried low confidence. In contrast, the metal complexes were consistently predicted not to be CYP2D6 substrates, suggesting greater metabolic stability and reduced enzymatic breakdown. Excretion, the predicted clearance values (CLtot) were higher for the metal complexes compared to the free ligands, as shown in Table S12, Supporting Information, suggesting that the complexes may be eliminated from the body more efficiently. Despite these differences in clearance, all compounds were predicted to have short half‐lives <3 h. The prediction confidence was generally high for the metal complexes and moderate to high for the ligands. Toxicity, the predictions showed that all ligands and complexes were considered toxic in both AMES mutagenicity and hERG channel inhibition models, with prediction probabilities ranging from 0.401 to 0.53, as shown in **Table** **3**. However, all results were assigned low‐confidence scores, suggesting uncertainty in model predictions and limited reliability, particularly for metal‐containing structures.

**Table 3:** Toxicity properties obtained for ADMET prediction.

	AMES Mutagenesis	Interpretation	hERG Blockers	Interpretation
Ligand 1	(0.492)	Low Confidence	(0.501)	Low Confidence
Ligand 2	(0.435)	Low Confidence	(0.492)	Low Confidence
Ligand 3	(0.401)	Low Confidence	(0.530)	Low Confidence
Complex 1	(0.488)	Low Confidence	(0.483)	Low Confidence
Complex 2	(0.459)	Low Confidence	(0.425)	Low Confidence
Complex 3	(0.527)	Low Confidence	(0.523)	Low Confidence

### 3.10 Anticancer Activity

**Figure 6:**
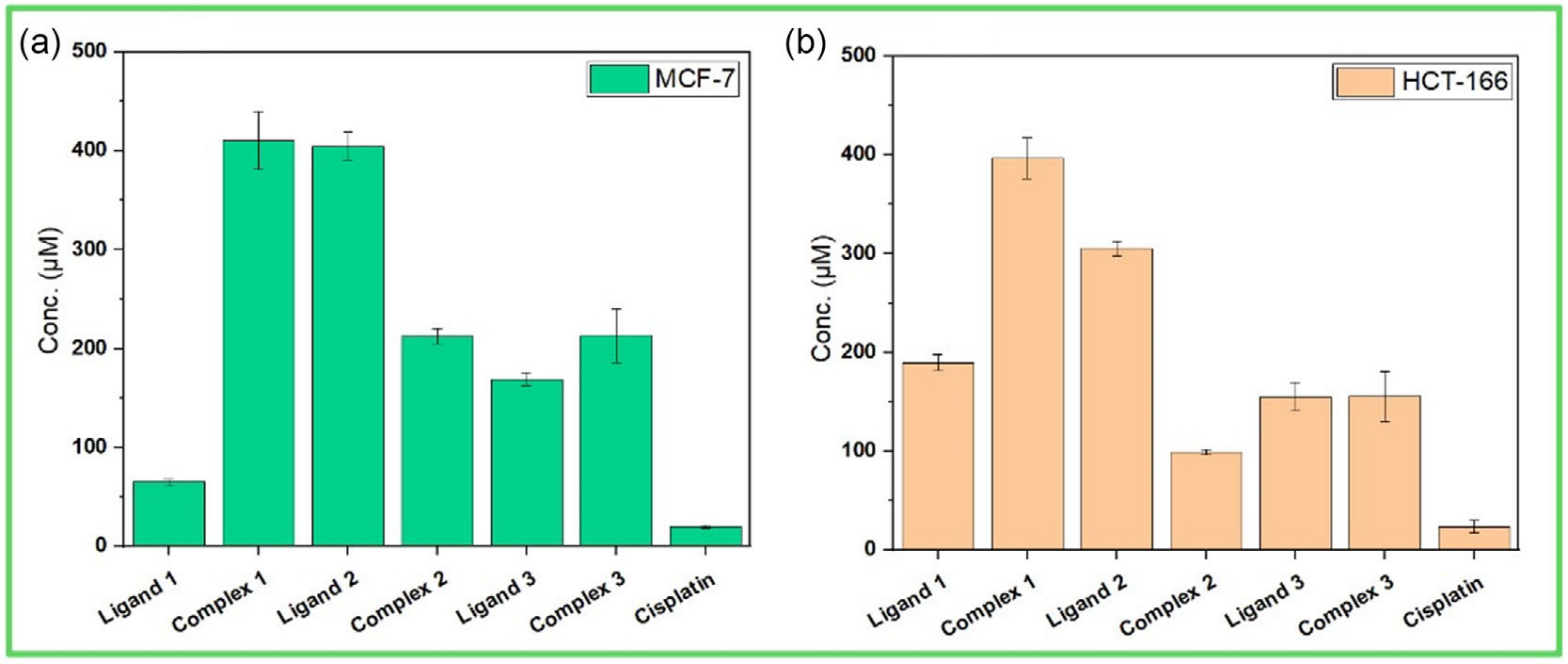
The IC_50_ for the ligands and their complexes (**1–3**) toward MCF‐7 and HCT‐116 cells.

**Figure 7:**
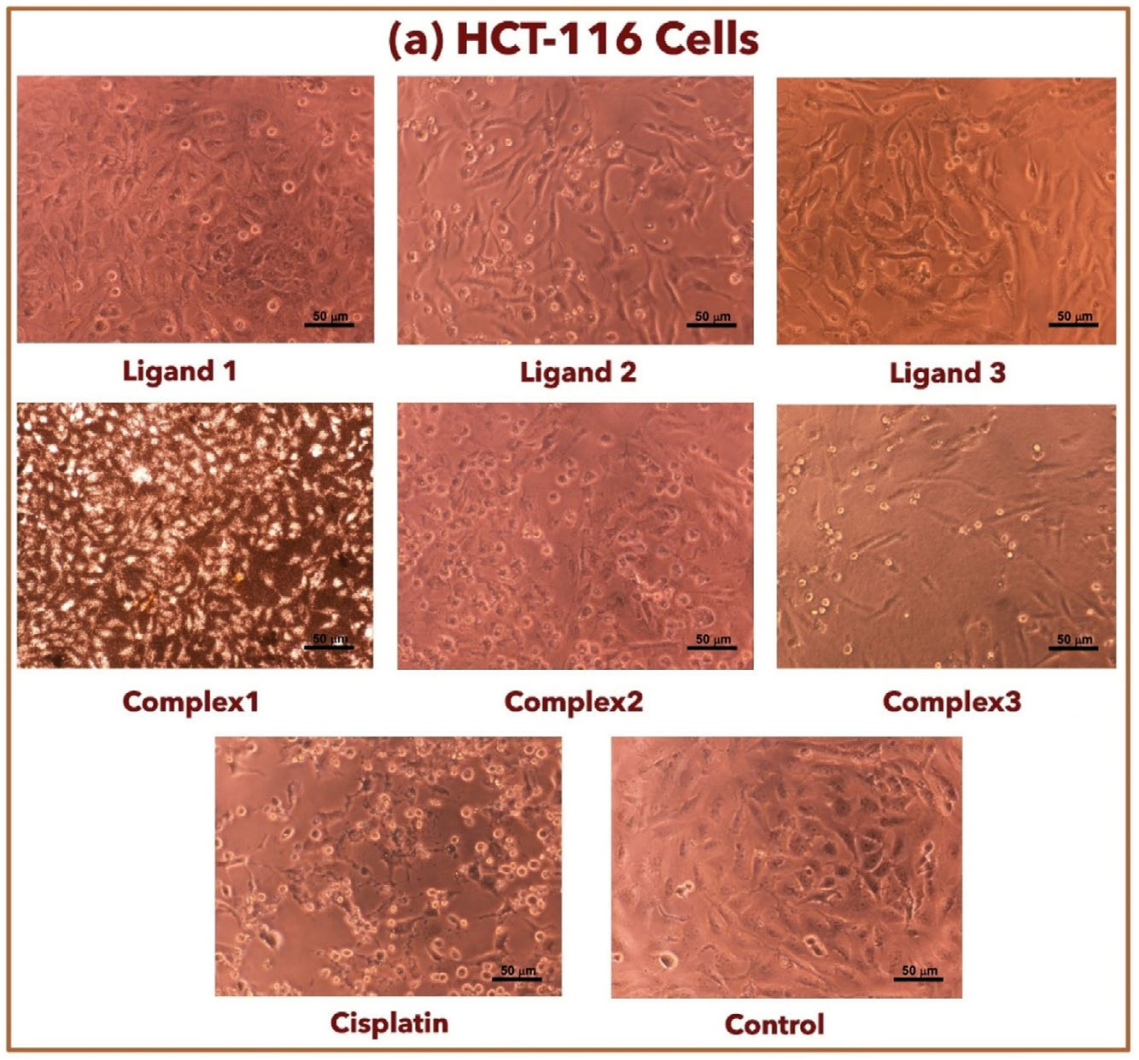
Optical microscope images of compounds toward the HCT‐116 cells.

**Figure 8:**
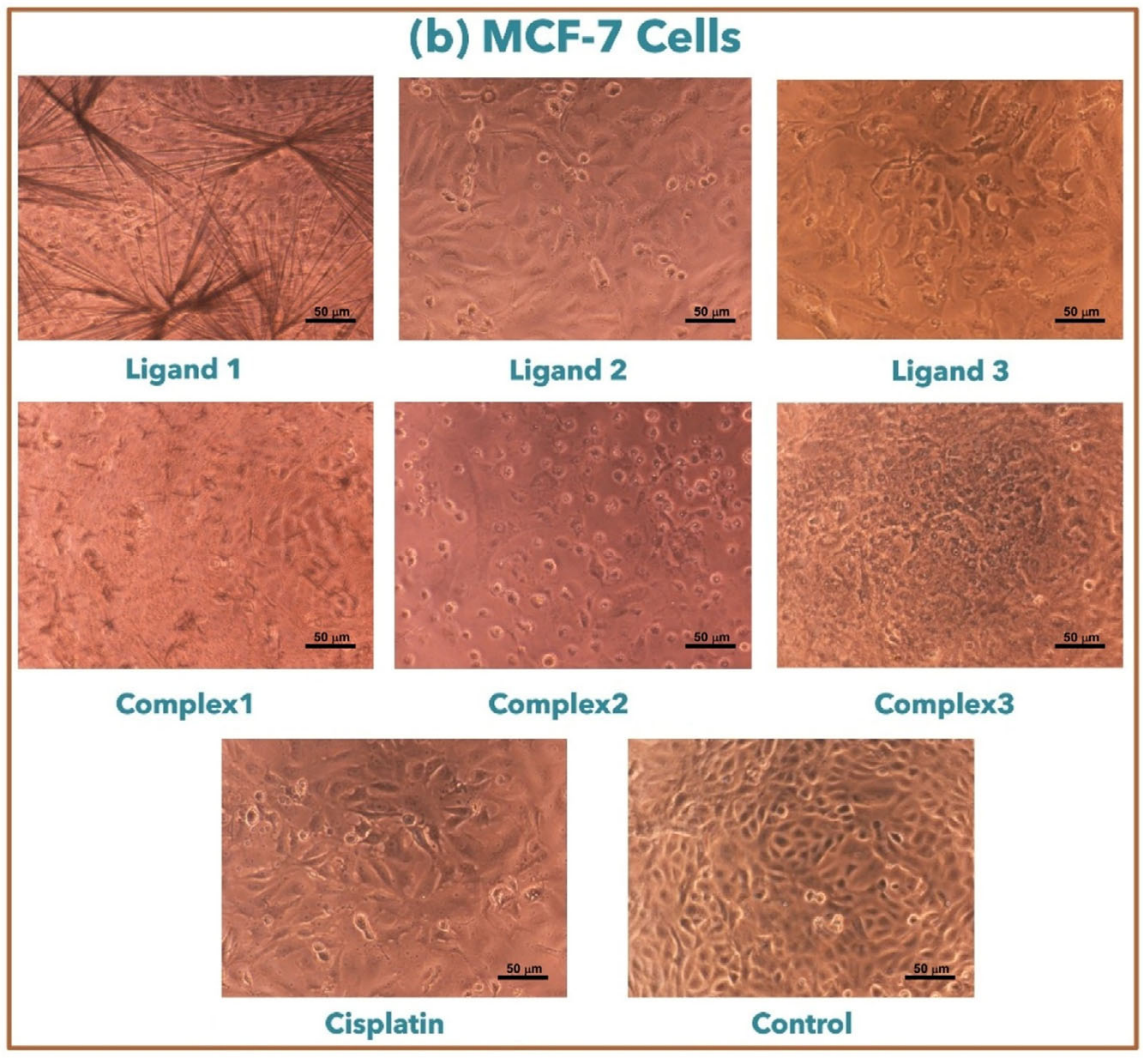
Optical microscope images for compounds against MCF‐7 cells.

The in vitro cytotoxic activity of salen‐type ligands and their complexes (**1**–**3**) was evaluated against MCF‐7 and HCT‐116 utilizing cisplatin as a reference, as shown in **Table** **4**. The test of compounds toward MCF‐7 cell lines showed that complex **2** has the highest activity (lower IC_50_) compared to its parent ligand **2**, as shown in **Figure** **6**a.^[^ ^30^ ^]^ The investigation of compounds toward HCT‐116 cell lines illustrated that complex **2** has a lower IC_50_ compared to its ligand **2**, as shown in Figure 6b. Among all compounds, complex (**2**) is considered the best behavior toward MCF‐7 and HCT‐116 cell lines with lower IC_50_ compared to other complexes (**1–3**) with parent ligands (**1–3**) as illustrated in **Figure** **7** and **8**.

**Table 4:** The values of IC_50_ of ligands and their copper(II) complexes against MCF‐7 and HCT‐116 cells.

Compounds	IC_50_			
	MCF‐7		HCT‐116	
	[μg mL^−1^]	[μM]	[μg mL^−1^]	[μM]
Ligand 1	20.61 ± 0.884	65.15 ± 2.794	60.01 ± 2.629	189.7 ± 8.310
Ligand 2	108.6 ± 3.782	404.7 ± 14.09	81.89 ± 2.053	305.2 ± 7.651
Ligand 3	60.25 ± 2.270	169.0 ± 6.369	55.29 ± 5.075	155.1 ± 14.24
Complex 1	170 ± 12.1	410.7 ± 29.23	164.2 ± 8.503	396.7 ± 20.54
Complex 2	77.76 ± 2.800	212.5 ± 7.653	36.19 ± 0.875	98.91 ± 2.391
Complex 3	96.89 ± 12.33	213.4 ± 27.16	70.55 ± 11.66	155.4 ± 25.68
Cisplatin	5.948 ± 0.608	19.82 ± 2.026	7.095 ± 2.066	23.65 ± 6.885

Different patterns show up when the inhibitory effects of complexes and ligands are compared independently on MCF‐7 and HCT‐116 cell lines. Considering the MCF‐7 cell line, for ligands: ligand **2** has weaker inhibitory effects with an IC_50_ value of 108.6 μg mL^−1^ (404.7 μm), while ligand **1** exhibits moderate inhibitory action on MCF‐7 cells with an IC_50_ value of 20.61 μg mL^−1^ (65.15 μm). Ligand **3**, with an IC_50_ value of 60.25 μg mL^−1^ (169.0 μm), exhibits a moderate inhibitory effect on MCF‐7 cells, falling between the two ligands **1** and **2**. For complexes: complex **1**'s presented IC_50_ value of 170 μg mL^−1^ (410.7 μm), suggesting that it has less inhibitory power over MCF‐7 cells. The MCF‐7 cells exhibit considerable inhibitory activity toward complex **2** and complex **3**. Complex 2's IC_50_ value is 77.76 μg mL^−1^ (212.5 μm), while complex **3**'s IC_50_ value is 96.89 μg mL^−1^ (213.4 μm).

Regarding the HCT‐116 cell line, ligand **1** exhibits moderate inhibitory effects, as indicated by its IC_50_ value of 60.01 μg mL^−1^ (189.7 μm). Conversely, ligands **2** and **3** display IC_50_ values of 81.89 μg mL^−1^ (305.2 μm) and 55.29 μg mL^−1^ (155.1 μm), respectively, indicating varying inhibitory levels. The complexes’ inhibitory effects on HCT‐116 cells differ from those of their individual ligands; complex **1** has a greater IC_50_ value than ligand **1**, complex **2** exhibits stronger inhibition than its parent ligand, and complex **3** has inhibitory effects that are comparable to ligand **3**.

When the complexes are compared to their parent ligands, complex **1** has a greater IC_50_ value than ligand **1** in both MCF‐7 and HCT‐116 cells, suggesting a possible decline in inhibitory action after complex formation. In contrast, complex **2** shows a much higher inhibitory impact than ligand **2**, indicating that complexation has increased potency. On the other hand, complex **3** shows similar inhibitory effects to ligand **3**, suggesting that the complexation process does not appreciably change the parent ligand's inhibitory activity.

**Figure 9:**
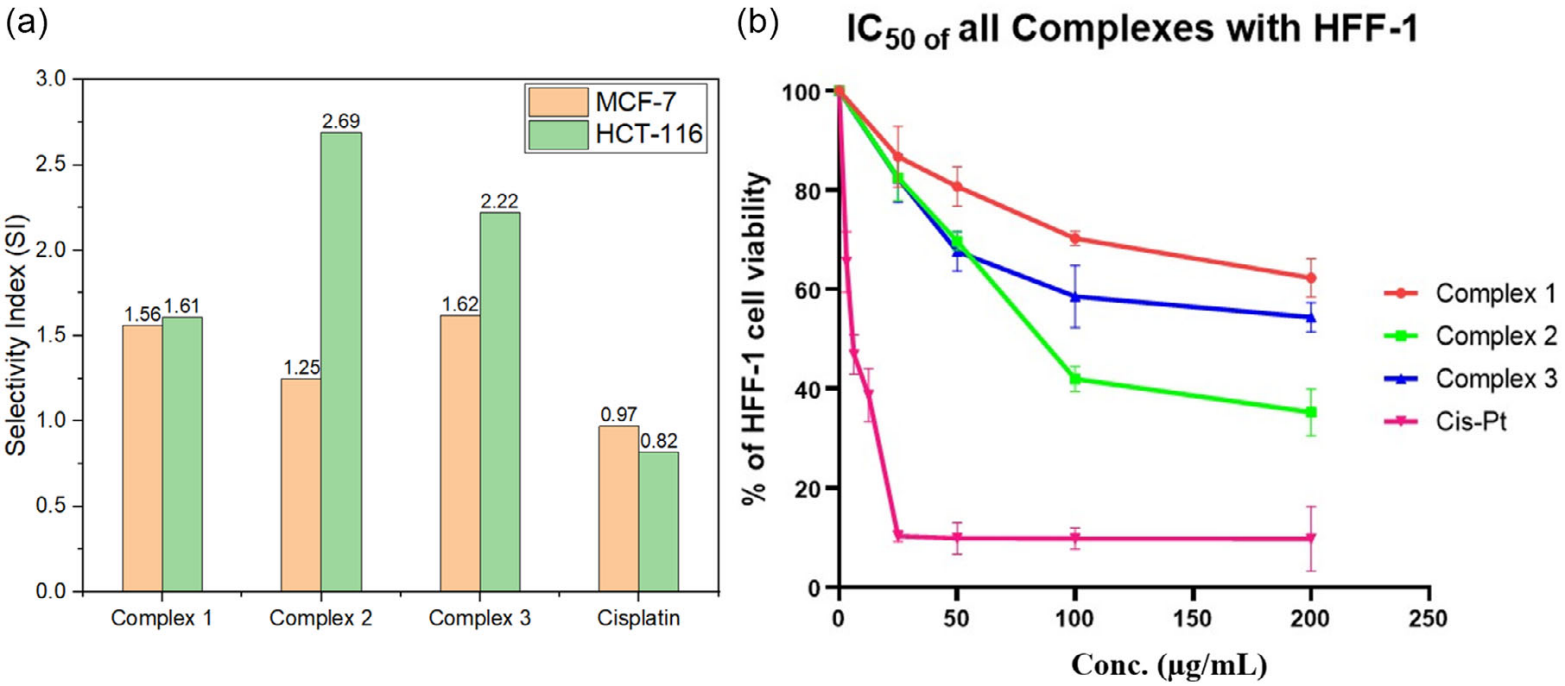
a) The SI of complexes (1–3) and cisplatin toward two cancer cell lines, MCF‐7 and HCT‐116. b) The cell viability percentage for copper(II) complexes compared to cisplatin.

**Figure 10:**
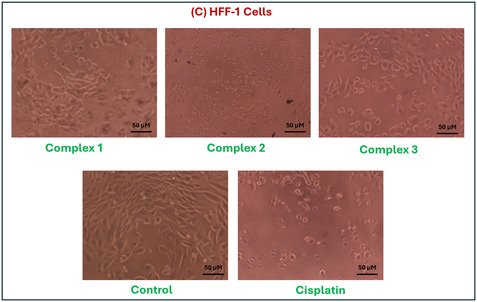
Optical microscope images of compounds against normal HFF‐1 cell lines.

Copper(II) complexes **1**–**3** were tested against normal HFF‐1 fibroblasts, as shown in **Table** **5**. Selectivity index (SI) demonstrated that complex **2** was the most selective, with an SI of 2.69 for HCT‐116. Complex **3** also showed favorable selectivity (SI = 2.22 for HCT‐116; 1.62 for MCF‐7), while complex **1** showed the lowest toxicity toward HFF‐1 (IC_50_ = 265.00 μg mL^−1^) with moderate selectivity, as illustrated in **Figure** **9** and **10**. In contrast, cisplatin presented poor selectivity (SI < 1), consistent with its known cytotoxicity in normal cells. These results highlight the potential of salen‐type copper(II) complexes, particularly complex **2**, as more selective alternatives to platinum‐based chemotherapy.^[^ ^51^ ^]^

**Table 5:** SI and IC_50_ values for copper(II) complexes (1–3) and Cisplatin against HFF‐1, MCF‐7 and HCT‐116 cells.

	HFF‐1	MCF‐7			HCT‐116		
	[μg mL^−1^]	[μg mL^−1^]	SIa)	[μm]	[μg mL^−1^]	SI	[μm]
Complex 1	265.00	170.00	1.56	410.70	164.20	1.61	396.70
Complex 2	97.18	77.76	1.25	212.50	36.19	2.69	98.91
Complex 3	156.90	96.89	1.62	213.40	70.55	2.22	155.40
Cisplatin	5.79	5.95	0.97	19.82	7.10	0.82	23.65

a) SI (Selectivity Index) = IC_50_ on normal cells (HFF‐1)/IC_50_ on cancer cells.

Although the copper(II) complexes exhibited higher IC_50_ values than cisplatin, they have significantly lower toxicity toward normal HFF‐1 fibroblasts, resulting in higher selectivity indexes. To complement the experimental results, ADMET predictions (Table S13, Supporting Information) were made, where all compounds were recognized as toxic in AMES and hERG models but with low‐confidence scores. This suggests limited predictive accuracy, especially for metal‐based compounds that fall outside conventional quantitative structure activity releationship (QSAR) model domains. The findings support the potential of the synthesized copper(II) complexes as safer alternatives to traditional platinum drugs and justify their continued investigation and structural refinement.

## 4 Conclusion

Three salen‐type ligands and their copper(II) complexes were synthesized and structurally characterized, and their anticancer potential was assessed through experimental and computational approaches. The spectroscopic analysis alongside the DFT studies confirmed the octahedral geometric structure of copper(II) complexes. Molecular docking studies against cancer targets (3eqm and 3ig7) illustrated complex **2** as the most favorable binder. In accordance with these findings, in vitro assays in MCF‐7 and HCT‐116 cancer cell lines showed moderate cytotoxic activity. Complex **2** presented the highest selectivity index, particularly toward HCT‐116. Although the IC_5__0_ values were higher than that of cisplatin, the copper(II) complexes demonstrated lower toxicity toward normal HFF‐1 fibroblasts, supporting a more favorable therapeutic index. Furthermore, in silico ADMET predictions suggested toxicity in AMES and hERG models; however, these results were accompanied by low confidence, highlighting the limitations of current predictive models for metal‐containing compounds. These data collectively highlight complex **2** as a promising structure for developing selective metal‐based anticancer agents with reduced off‐target toxicity.

## Conflict of Interest

The authors declare no conflict of interest.

## Author Contributions

**Abdellatif A. Helaly:** conceptualization (supporting); data curation (lead); formal analysis (lead); investigation (lead); methodology (lead); software (equal); visualization (lead); writing—original draft (lead); writing—review & editing (lead). **Bandar A. Babgi**: conceptualization (lead); formal analysis (lead); supervision (lead); writing—review & editing (lead). **Yoji Kobayashi**: conceptualization (equal); formal analysis (lead); supervision (lead); writing—review & editing (lead). **Rohit K. Rai:** investigation (supporting). **Ehab M. M. Ali**: investigation (supporting); methodology (supporting); writing—review & editing (supporting). **Abdulaziz A. Kalantan**: methodology (supporting). **Walid M. I. Hassan:** software (lead); visualization (supporting). **Mostafa A. Hussien**: conceptualization (lead); formal analysis (lead); supervision (lead); writing—review & editing (lead). **Muhammad M. I. Ismail**: software (supporting).

## Supporting information

Supplementary Material

## Data Availability

The data that support the findings of this study are available in the supplementary material of this article.

## References

[1] A. Soroceanu , A. Bargan , Crystals 2022, 12, 1436.

[2] K. Mondal , S. Mistri , Comments Inorg. Chem. 2023, 43, 77.

[3] Q. U. A. Sandhu , M. Pervaiz , A. Majid , U. Younas , Z. Saeed , A. Ashraf , R. R. M. Khan , S. Ullah , F. Ali , S. Jelani , J. Coord. Chem. 2023, 76, 1094.

[4] B. Es‐Sounni , A. Nakkabi , A. Bouymajane , I. Elaaraj , M. Bakhouch , F. R. Filali , M. El Yazidi , N. El Moualij , M. Fahim , Biointerface Res. Appl. Chem. 2023, 13, 333.

[5] P. S. Steinlandt , L. Zhang , E. Meggers , Chem. Rev. 2023, 123, 4764. 36988612 10.1021/acs.chemrev.2c00724 PMC10141360

[6] I. Pietro Oliveri , S. Di Bella , Chemistry 2023, 5, 119.

[7] V. Iannace , F. Sabaté , M. Bartlett , J. Berrones Reyes , A. Lázaro , A. Fantoni , R. Vilar , L. Rodríguez , A. Dalla Cort , Eur. J. Inorg. Chem. 2023, 26, e202300144.

[8] W. Tian , W. Zhong , Z. Yang , L. Chen , S. Lin , Y. Li , Y. Wang , P. Yang , X. Long , J. Inorg. Biochem. 2024, 251, 112434. 38029537 10.1016/j.jinorgbio.2023.112434

[9] D. Majumdar , S. Roy , J. Elizabeth Philip , B. Tüzün , S. Hazra , Inorg. Chem. Commun. 2024, 160, 111933.

[10] O. A. Zalevskaya , Y. A. Gur'eva , Russ. J. Coord. Chem. 2021, 47, 861.

[11] M. Claudel , J. V. Schwarte , K. M. Fromm , Chemistry 2020, 2, 849.

[12] J. Gao , Y. G. Liu , Y. Zhou , R. A. Zingaro , ChemMedChem 2007, 2, 1723. 17943711 10.1002/cmdc.200700049

[13] J. C. Pessoa , I. Correia , Coord. Chem. Rev. 2019, 388, 227.

[14] R. C. Pratt , C. T. Lyons , E. C. Wasinger , T. D. P. Stack , J. Am. Chem. Soc. 2012, 134, 7367. 22471355 10.1021/ja211247f PMC3343640

[15] A. Erxleben , Inorg. Chim. Acta 2018, 472, 40.

[16] X. Q. Zhou , Y. Li , D. Y. Zhang , Y. Nie , Z. J. Li , W. Gu , X. Liu , J. L. Tian , S. P. Yan , Eur. J. Med. Chem. 2016, 114, 244. 26994692 10.1016/j.ejmech.2016.02.055

[17] R. Kunert , C. Philouze , F. Berthiol , O. Jarjayes , T. Storr , F. Thomas , Dalton Trans. 2020, 49, 12990. 32909589 10.1039/d0dt02524k

[18] H. A. Kiwaan , A. S. El‐Mowafy , A. A. El‐Bindary , J. Mol. Liq. 2021, 326, 115381.

[19] OriginPro (Version 2024), Learning Edition, OriginLab Corporation, Northampton, MA.

[20] A. Altomare , C. Cuocci , C. Giacovazzo , A. Moliterni , R. Rizzi , N. Corriero , A. Falcicchio , J. Appl. Crystallogr. 2013, 46, 1231.

[21] C. Altenbach , D. Budil , Appl. Magn. Reson. 2024, 55, 159.

[22] G. M. Morris , H. Ruth , W. Lindstrom , M. F. Sanner , R. K. Belew , D. S. Goodsell , A. J. Olson , J. Comput. Chem. 2009, 30, 2785. 19399780 10.1002/jcc.21256 PMC2760638

[23] Y. Myung , A. G. C. De Sá , D. B. Ascher , Nucleic Acids Res. 2024, 52, W469. 38634808 10.1093/nar/gkae254 PMC11223837

[24] N. E. Alshaikh , M. Zaki , A. A. Sharfalddin , N. S. Al‐Radadi , M. A. Hussien , W. M. I. Hassan , Arabian J. Chem. 2023, 16, 104845.

[25] E. M. M. Ali , A. A. Elashkar , H. Y. El‐Kassas , E. I. Salim , Int. J. Biol. Macromol. 2018, 120, 1170. 30172815 10.1016/j.ijbiomac.2018.08.118

[26] S. P. Langdon , in Cancer Cell Culture: Methods in Molecular Medicine, Vol. 88 (Ed: S. P. Langdon ), Humana Press 2004, 10.1385/1-59259-406-9:3.

[27] E. M. Abdalla , L. H. Abdel Rahman , A. A. Abdelhamid , M. R. Shehata , A. A. Alothman , A. Nafady , Appl. Organomet. Chem. 2020, 34, e5912.

[28] L. Tong , Y. F. Ding , X. Li , L. L. Man , W. K. Dong , J. Coord. Chem. 2023, 76, 1635.

[29] A. J. Jarad , M. A. Dahi , T. H. Al‐Noor , M. M. El‐ajaily , S. R. AL‐Ayash , A. Abdou , J. Mol. Struct. 2023, 1287, 135703.

[30] A. Helaly , H. Sahyon , H. Kiwan , A. G. Shoair , A. El‐Bindary , Biointerface Res. Appl. Chem. 2023, 13, 365.

[31] N. George , G. Singh , R. Singh , G. Singh , Priyanka , H. Singh , G. Kaur , J. Singh , J. Mol. Struct. 2023, 1288, 135666.

[32] M. M. Gaafar , F. M. Eltaweel , H. A. Fouda , M. Y. Abdelaal , J. Bioact. Compat. Polym. 2022, 37, 359.

[33] D. Marcinkowski , A. Adamski , M. Kubicki , G. Consiglio , V. Patroniak , T. Ślusarski , M. Açıkgöz , D. Szeliga , N. Vadra , M. Karbowiak , I. Stefaniuk , C. Rudowicz , A. Gorczyński , M. Korabik , Dalton Trans. 2022, 51, 12041. 35876304 10.1039/d2dt01564a

[34] E. Garribba , G. Micera , J. Chem. Educ. 2006, 83, 1229.

[35] A. Z. El‐Sonbati , M. A. Diab , A. E. B. El‐Bindary , A. F. Shoair , M. A. Hussein , R. A. El‐Boz , J. Mol. Struct. 2017, 1141, 186.

[36] A. Oral Sarıoğlu , Polyhedron 2024, 260, 117099.

[37] B. Rogalewicz , A. Climova , E. Pivovarova , J. Sukiennik , M. Szczesio , K. Gas , M. Sawicki , K. Czarnecka , P. Szyma , M. Pitucha , A. Czylkowska , Molecules 2022, 27, 2703. 35566053 10.3390/molecules27092703 PMC9100868

[38] M. J. Frisch , G. W. Trucks , H. B. Schlegel , G. E. Scuseria , M. A. Robb , J. R. Cheeseman , G. Scalmani , V. Barone , G. A. Petersson , H. Nakatsuji , X. Li , M. Caricato , A. V. Marenich , J. Bloino , B. G. Janesko , R. Gomperts , B. Mennucci , H. P. Hratchian , J. V. Ortiz , A. F. Izmaylov , J. L. Sonnenberg , D. Williams‐Young , F. Ding , F. Lipparini , F. Egidi , J. Goings , B. Peng , A. Petrone , T. Henderson , D. Ranasinghe , et al., Gaussian 09 Software 2016.

[39] K. P. Manoj , N. Elangovan , S. Chandrasekar , Inorg. Chem. Commun. 2022, 139, 109324.

[40] L. Q. Chai , X. F. Zhang , L. J. Tang , J. Mol. Struct. 2021, 1245, 131028.

[41] W. H. Mahmoud , R. G. Deghadi , G. G. Mohamed , J. Organomet. Chem. 2020, 917, 121113.

[42] O. A. El‐Gammal , A. A. El‐Bindary , F. Sh , J. Mol. Liq. 2022, 346, 117850.

[43] M. H. Abdel‐Rhman , R. Motawea , A. Belal , N. M. Hosny , J. Mol. Struct. 2022, 1251, 131960.

[44] O. A. El‐Gammal , F. S. Mohamed , G. N. Rezk , A. A. El‐Bindary , J. Mol. Liq. 2021, 330, 115522.

[45] R. N. Asha , M. Sankarganesh , N. Bhuvanesh , B. R. D. Nayagam , J. Mol. Struct. 2022, 1250, 131692.

[46] R. Huey , G. M. Morris , A. J. Olson , D. S. Goodsell , J. Comput. Chem. 2007, 28, 1145. 17274016 10.1002/jcc.20634

[47] S. H. S. Saleem , M. Sankarganesh , J. D. Raja , P. R. A. Jose , A. Sakthivel , T. C. Jeyakumar , R. N. Asha , J. Saudi Chem. Soc. 2021, 25, 101225.

[48] F. Y. Alomari , A. A. Sharfalddin , M. H. Abdellattif , D. Domyati , A. S. Basaleh , M. A. Hussien , Molecules 2022, 27, 649. 35163913 10.3390/molecules27030649 PMC8838224

[49] G. Venkatesh , P. Vennila , S. Kaya , S. Ben Ahmed , P. Sumathi , V. Siva , P. Rajendran , C. Kamal , ACS Omega 2024, 9, 8123. 38405527 10.1021/acsomega.3c08526 PMC10882688

[50] B. El Bali , A. Direm , M. Lachkar , D. Díaz‐García , S. Gómez‐Ruiz , H. Dihazi , Transition Met. Chem. 2024, 49, 465.

[51] S. Veschi , S. Carradori , L. De Lellis , R. Florio , D. Brocco , D. Secci , P. Guglielmi , M. Spano , A. P. Sobolev , A. Cama , J. Enzyme Inhib. Med. Chem. 2020, 35, 1331. 32588672 10.1080/14756366.2020.1780228 PMC7470072

