# Supplementary material for: Salen‐Type Copper(II) Complexes: Synthesis, Characterization, Computational Studies, Molecular Docking, Anticancer Potential, and Pharmacokinetic Prediction
Source: ChemistryOpen. 2025 Jul 1;14(9):e202500061. doi: 10.1002/open.202500061 (PMC12409841; doi:10.1002/open.202500061)
Supplement: Supplementary file 1 — Supplementary Material [file OPEN-14-e202500061-s001.pdf]

Supplementary Materials

Salen-type Copper(II) Complexes: Synthesis, Characterization, Computational Studies, Molecular Docking, Anticancer Potential, and Pharmacokinetic Prediction.

Abdellatif A. Helaly [a b], Bandar A. Babgi [a], Yoji Kobayashi* [c], Rohit K. Rai [c], Ehab M. M. Ali [d e], Abdulaziz A. Kalantan [d], Walid M.I. Hassan [a], Mostafa A. Hussien* [a f], Muhammad M. I. Ismail [g h]

[a] Department of Chemistry, Faculty of Science, King Abdulaziz University (KAU), Jeddah 21589, Saudi Arabia
[b] Department of Chemistry, Faculty of Science, Damietta University, Damietta 34517, Egypt
[c] Center of Renewable Energy and Storage Technologies (CREST), Chemistry Program, Division of Physical Sciences and Engineering, King Abdullah University of Science and Technology (KAUST), Thuwal, 23955–6900, Saudi Arabia
[d] Department of Biochemistry, Faculty of Science, King Abdulaziz University (KAU), Jeddah 21589, Saudi Arabia
[e] Department of Chemistry, Faculty of Science, Tanta University, Tanta 31527, Egypt
[f] Department of Chemistry, Faculty of Science, Port Said University, Port Said 42521, Egypt
[g] Department of Physics, Faculty of Science, Port Said University, Port Said 42521, Egypt
[h] Department of Physics, Faculty of Science, Al-Baha University, Al-Baha 65799, Saudi Arabia

*Corresponding Authors: maabdulaal@kau.edu.sa and yoji.kobayashi@kaust.edu.sa

2. Experimental Section

S2.5. ADMET Prediction Workflow

The ADMET prediction workflow covered all major pharmacokinetic and toxicity categories. For **absorption:** *Caco-2 Permeability & Human Intestinal Absorption (HIA):* Predicted values (e.g., log Papp and absorption percentages) were obtained to estimate oral uptake efficiency. *Bioavailability:* Probabilities for achieving 20–50% bioavailability were calculated for both free ligands and copper(II) complexes. **Distribution:** *Blood–Brain Barrier (BBB) Penetration:* The potential for central nervous system access was estimated. *Plasma Protein Binding (PPB) and Volume of Distribution (VDss):* These endpoints provided insight into the extent and nature of tissue distribution. For **metabolism:** Predictive models assessed the likelihood of each compound acting as a substrate or inhibitor for key cytochrome P450 isoforms, including CYP3A4 and CYP2D6. **Excretion:** *Clearance (CLtot) and Half-life (t½):* The predicted clearance rates and half-lives were compared between ligands and their complexes. Finally, **toxicity:** Endpoints such as Ames mutagenesis, hERG inhibition, and hepatotoxicity (DILI) were modeled to assess potential safety concerns.

S2.7. In vitro anticancer activity

The in vitro cytotoxicity assay of compounds was investigated on human breast (MCF-7) and human colon (HCT-116) cells, and normal cell HFF-1 that were provided by the Tissue Culture Unit at the Biochemistry Department at King Abdulaziz University. The cells with complete Dulbecco's Modified Eagle's Medium (DMEM) containing 10% bovine fetal albumin and 1 % antibiotic (penicillin/streptomycin) (obtained from Life Technologies Gibco), cancer cell lines were grown. The flasks were incubated in sterile 5% CO_2 at 37°C and 95% humidity for 24-48 hours. Once 90% of the confluent cells were attached, 4 mL of 0.25% trypsin-EDTA solution was added, and the cells were then incubated in a CO_2 incubator for 5 minutes. Five milliliters of the whole medium were added to terminate the trypsin process. Pellets of cells were obtained after centrifugation for 5 min at 1500 rpm. The cells were suspended in the complete medium [1].

The number of cells was assigned with a hemocytometer and counted in the four major squares after staining 20 μL of this cell-containing media with 20 μL of 0.4% trypan blue. 100 μL of 10^4 cells suspended in complete media were added to each well of a 96-well microplate, and the plate was incubated for 24 h. For each compound, different concentrations (12.5, 25, 50, 100, and 200 μg/mL) were prepared, and each concentration containing media was added to the well after 24 hours of incubating the plate in a CO_2 incubator. Each concentration was replicated three times. The incubation time for the plate was 48 h after treatment. In each well, the medium was exchanged with 100 mL of 0.5 mg MTT/mL free media for 4 hours in the incubator. Each well was filled with 100 mL DMSO and incubated at room temperature for 15 min before being observed using a Bio-RAD microplate reader at 595 nm. The half-maximal inhibitory concentrations (IC_{50}) of the complexes were calculated from the curve of the percentage of cell viability versus different concentrations of the compounds, employing GraphPad Prism 9 software [2].

3. Results and discussion

S3.4. Nuclear magnetic resonance (NMR):

Ligand (1), as shown in **Figures S5** and **S6**, ^{1}HNMR data illustrates that there are two singlet peaks that appear at 8.43 and 10.79 ppm, which contribute to protons in HC=N and OH, respectively. The aromatic hydrogen peaks appear at 6.7, 6.82, 7.01 and 7.18 ppm with multiplet splitting. **^{13}C NMR** data demonstrated that two significant peaks appear at 161.24 and 163.79 ppm related to C=N and C-OH groups, respectively.

Ligand (2), as shown in **Figures S7** and **S8**, **^{1}HNMR** data illustrated that there are two singlet peaks appearing at 3.93 and 8.34 ppm which contribute to protons in bridge-CH_2 and HC=N respectively. The aromatic hydrogen peaks appear at 6.82, 6.96, 7.21 and 7.26 ppm with Multiplet splitting. **^{13}CNMR** data demonstrated that three important peaks at 59.64, 161.07 and 166.59 ppm related to bridge-CH_2, C=N and C-OH groups respectively.

Ligand (3), as shown in **Figures S9** and **S10**, **^{1}HNMR** data illustrates that there are three singlet peaks that appear at 3.96, 8.34 and 13.63 ppm which contribute to protons in bridge-CH_2, HC=N and OH respectively. The aromatic hydrogen peaks appear at 6.78, 6.84, 6.91 and 7.29 ppm with Multiplet splitting. Additionally, there is one triplet peak at 1.46 ppm related to aliphatic CH_3 and one quartet peak that appears at 4.12 for ethoxy CH_2. **^{13}C NMR** data demonstrated that four important peaks at 14.91, 59.64, 151.51 and 166.59 ppm related to aliphatic CH_3, bridge-CH_2, C-OH and C=N groups respectively.

S3.5. Thermal gravimetric analysis (TGA)

Ligand **1** has decomposed into two steps: the first step occurs at 196-327 °C due to the loss of $C_6H_{10}N_2O_2$ (found 44.63 %, Calc. 44.93 %), the second step occurs at 327-683 °C due to the loss of $C_{13}H_6$ (found 51.79 %, Calc. 51.26 %) and it has one carbon atom over 683 °C (found 3.58 %, Calc. 3.81 %). Ligand **2** has lost most of its weight percentage (found 91.61 %, Calc. 91.05 %) between 160 and 322 °C with a small Wt. % (found 8.39, Calc. 8.95) as residue over 322 °C due to C_2. Ligand **3** lost 61.8 Wt. % at 199-321 °C due to $C_9H_{20}N_2O_4$ but lost 38.2 Wt.% at 321-640 because of $C_{11}H_4$.

S3.6. PXRD

Figure S12 shows the X-ray diffraction pattern of the prepared complexes. The several diffraction peaks confirmed the polycrystalline nature of the prepared complexes. As mentioned previously, EXPO2014 software

was used to determine crystal scheme, space group, and lattice parameters of the prepared complexes, see **Table S6**. For complex **1**, Miller indices were assigned on a triclinic crystal of a temporary space group assignment as triclinic (P-1) with lattice parameters (a = 21.92 Å, b = 9.96 Å, c = 8.52 Å, α = 107.12°, β = 98.57°, γ = 82.36°). While in complex **2**, Miller indices were assigned on a temporary space group assignment as tetragonal (P4/m) with lattice parameter (a = 6.27 Å, b = 6.27 Å, c = 27.12 Å, and α = β = γ = 90°). Finally, complex **3**, Miller indices assigned on a temporary space group assignment as orthorhombic (Pmmm) with lattice parameter (a = 12.8 Å, b = 19.8 Å, c = 7.63 Å, α = β = γ = 90°). Moreover, the average crystallite size C_S for the prepared complexes were estimated using the Debye-Scherrer equation, **equation 1** [3]:

$$C_S = \frac{0.9\lambda}{\beta\ cos\theta} \qquad (1)$$

where λ is the wavelength, which is 0.15406 nm, θ is the half diffraction angle of 2θ, and β is the full width at half-maximum (FWHM) value of the XRD diffraction peaks. All peaks were used to calculate the average crystallite size (C_S) that were listed in **Table S6**

Figures

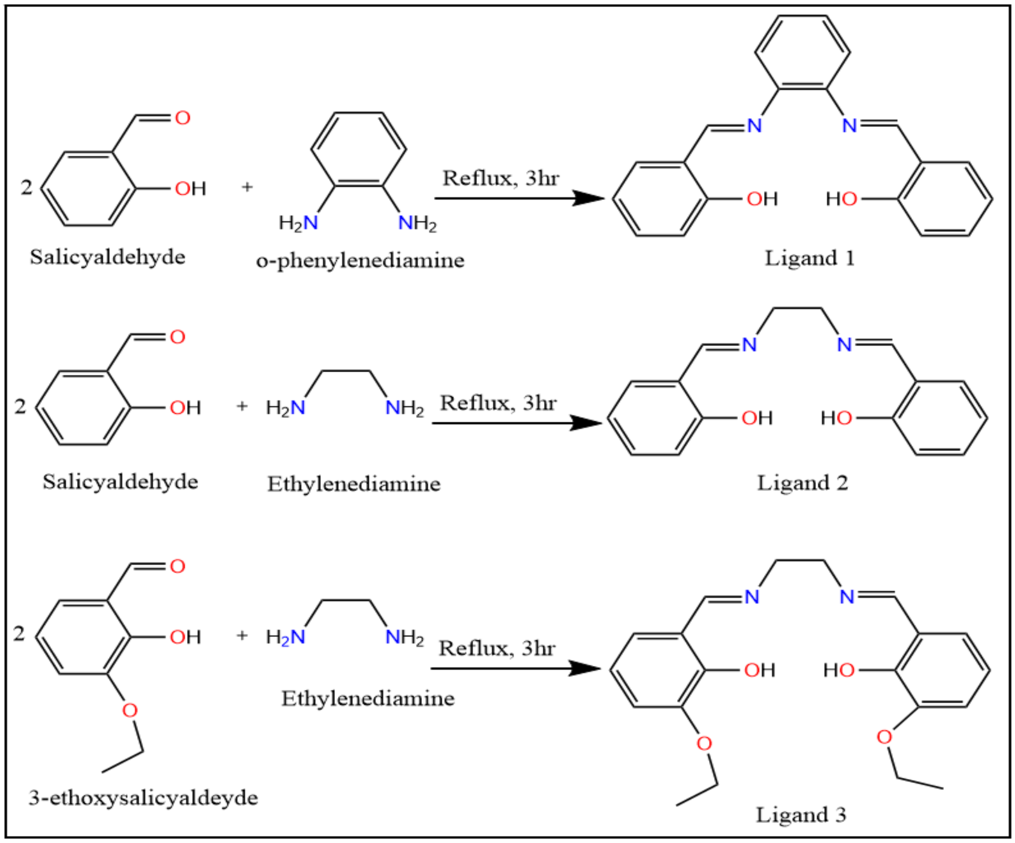

Scheme S1. Synthesis of Salen and salophen ligands

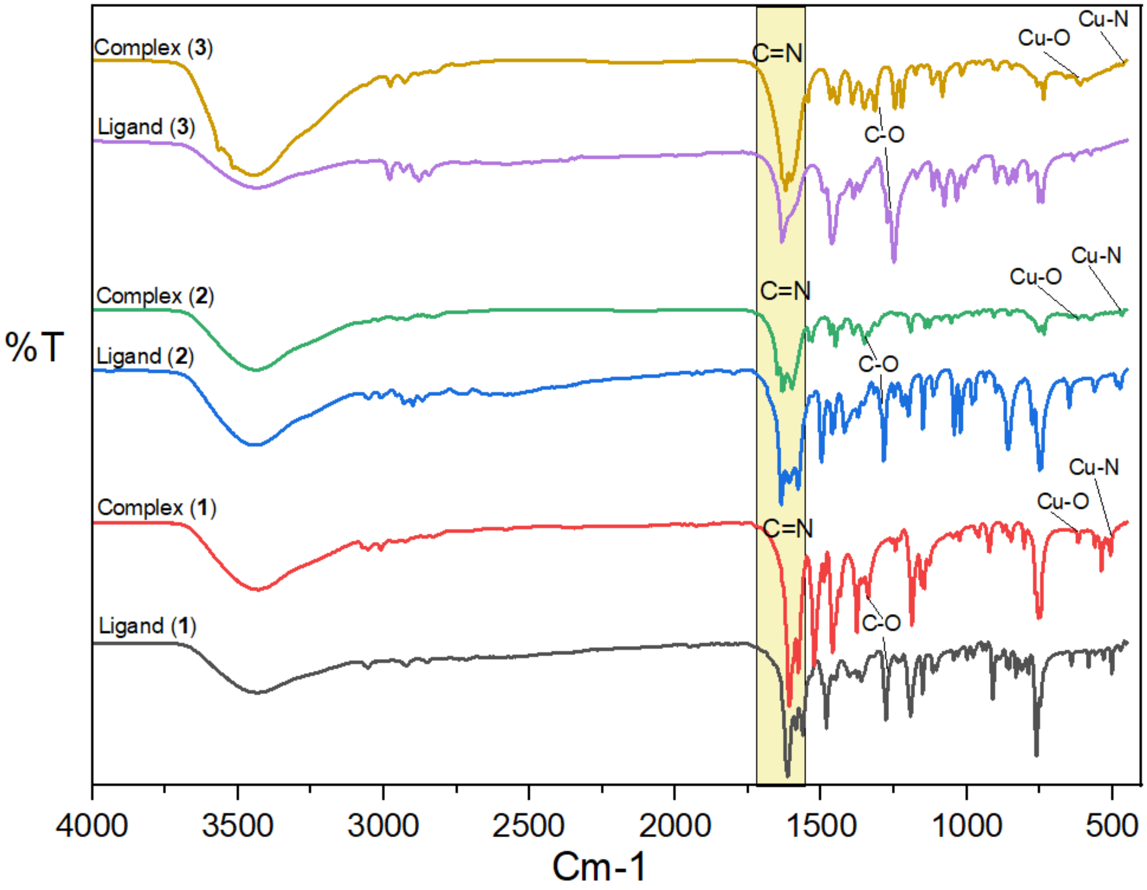

Figure S1. FTIR spectra of ligands and their complexes

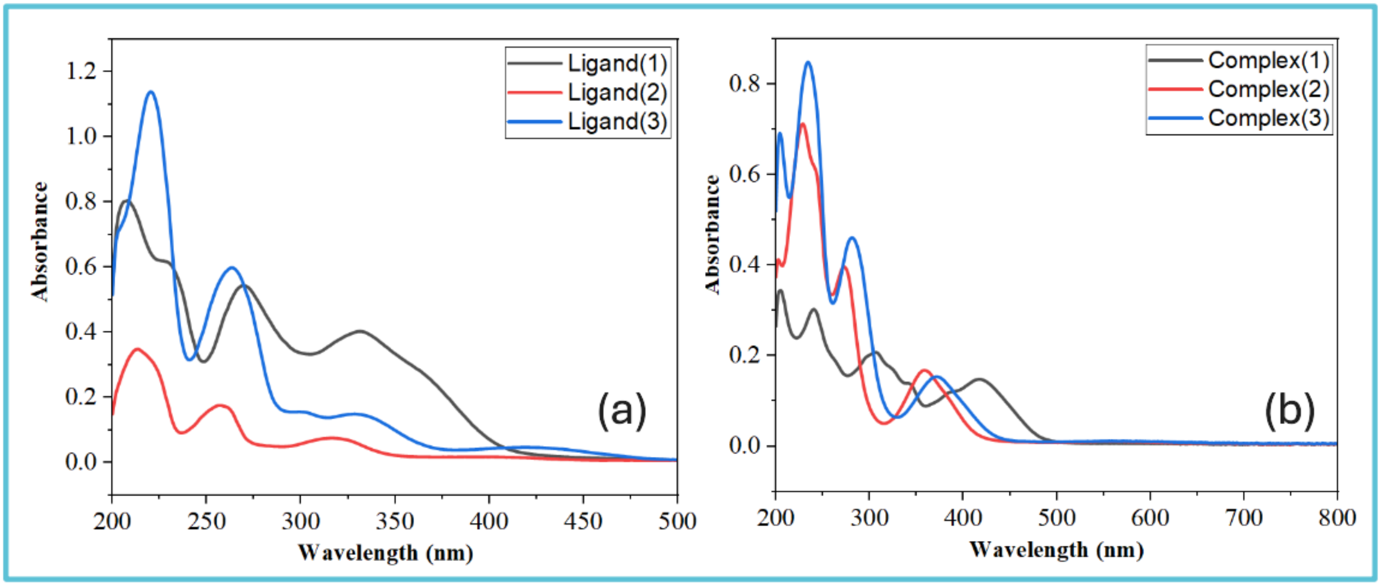

Figure S2. The optical absorption spectra of (a) ligands and (b) the copper(II) complexes.

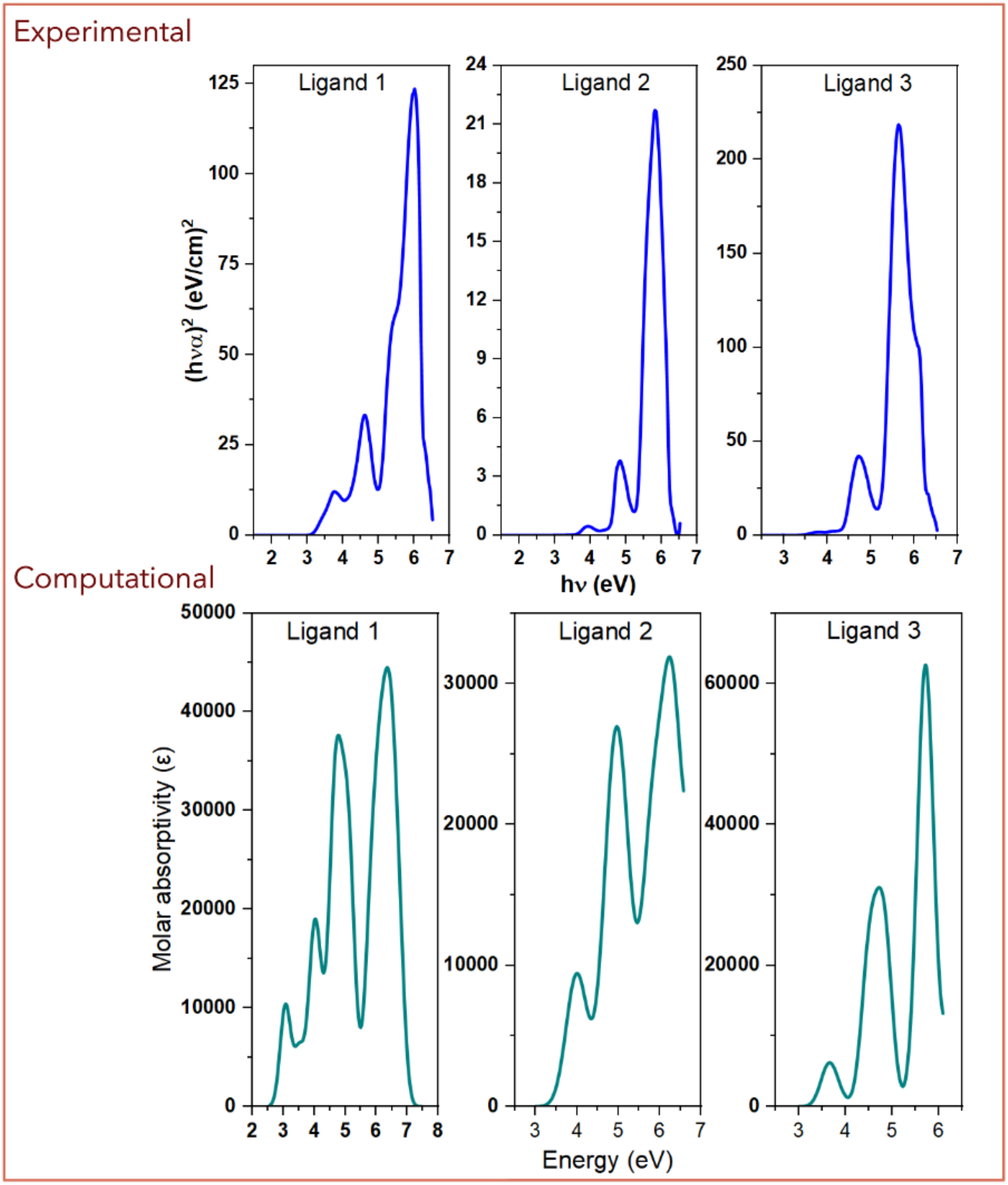

Figure S3. Calculated and measured optical absorption spectra of ligands (**1**-**3**).

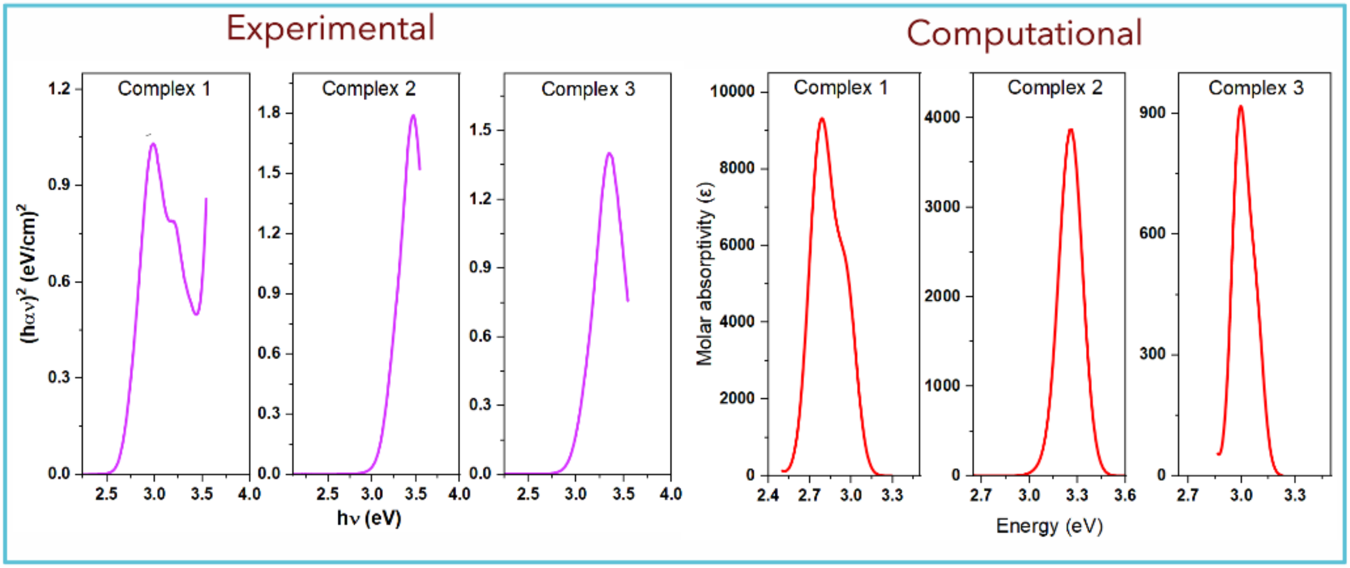

Figure S4. Calculated and measured optical absorption spectra of complexes (**1**-**3**).

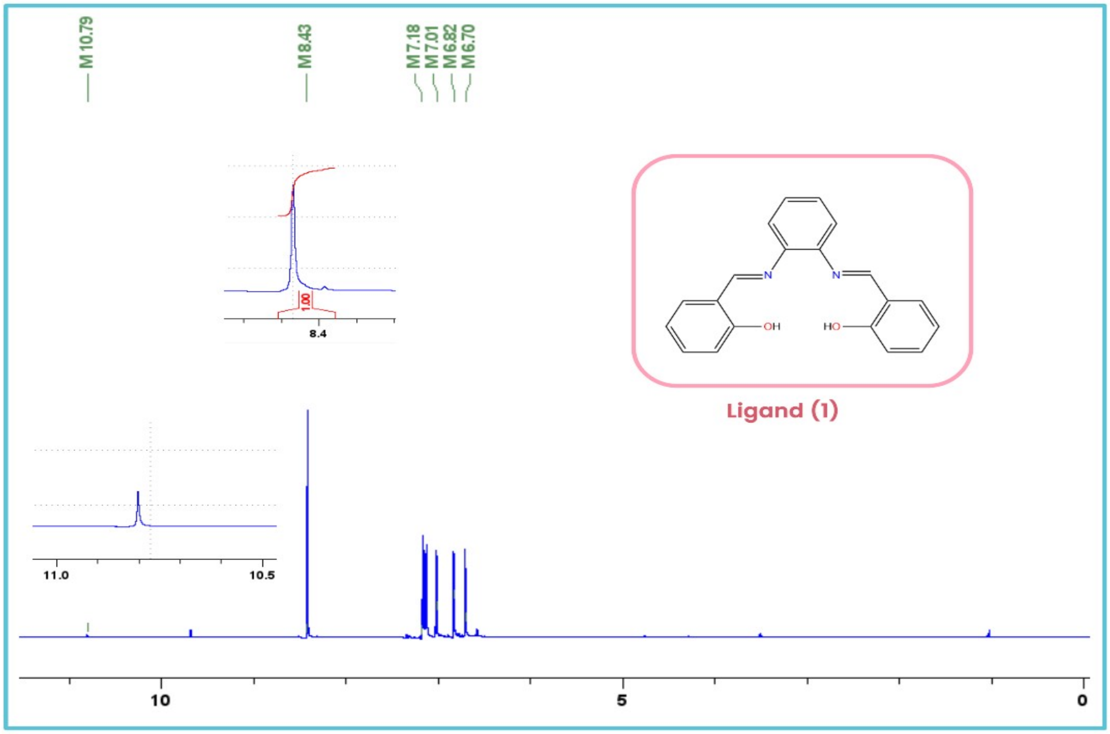

Figure S5. ^{1}HNMR spectrum of ligand (1).

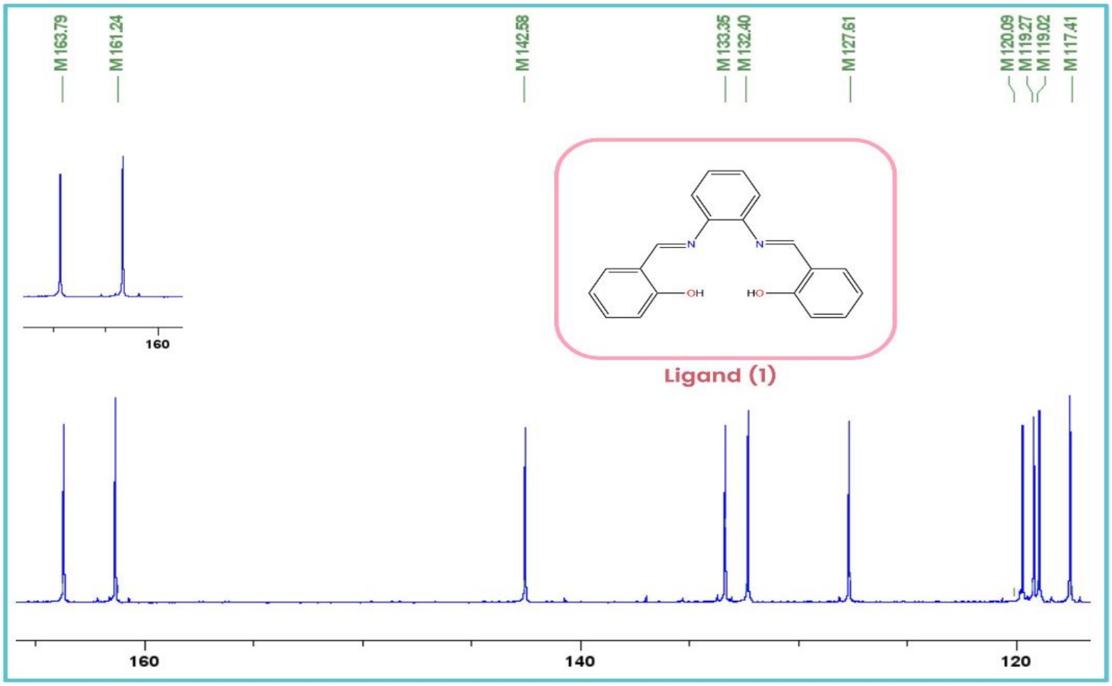

Figure S6. ^{13}CNMR spectrum of ligand (1).

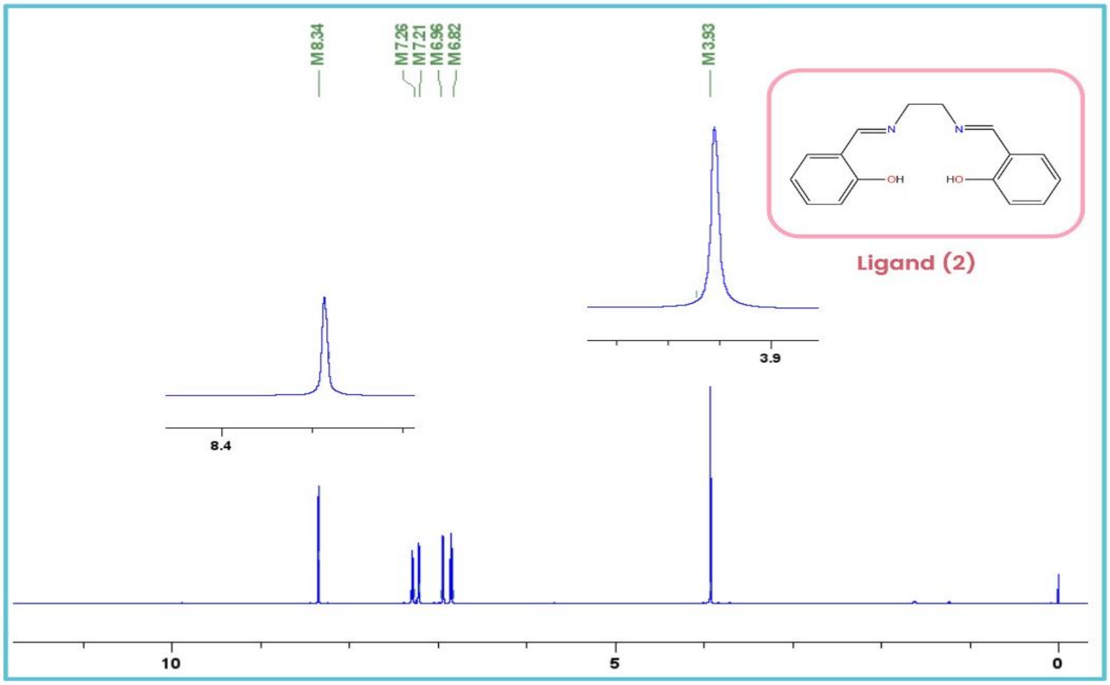

Figure S7. ^{1}H NMR spectrum of ligand (**2**).

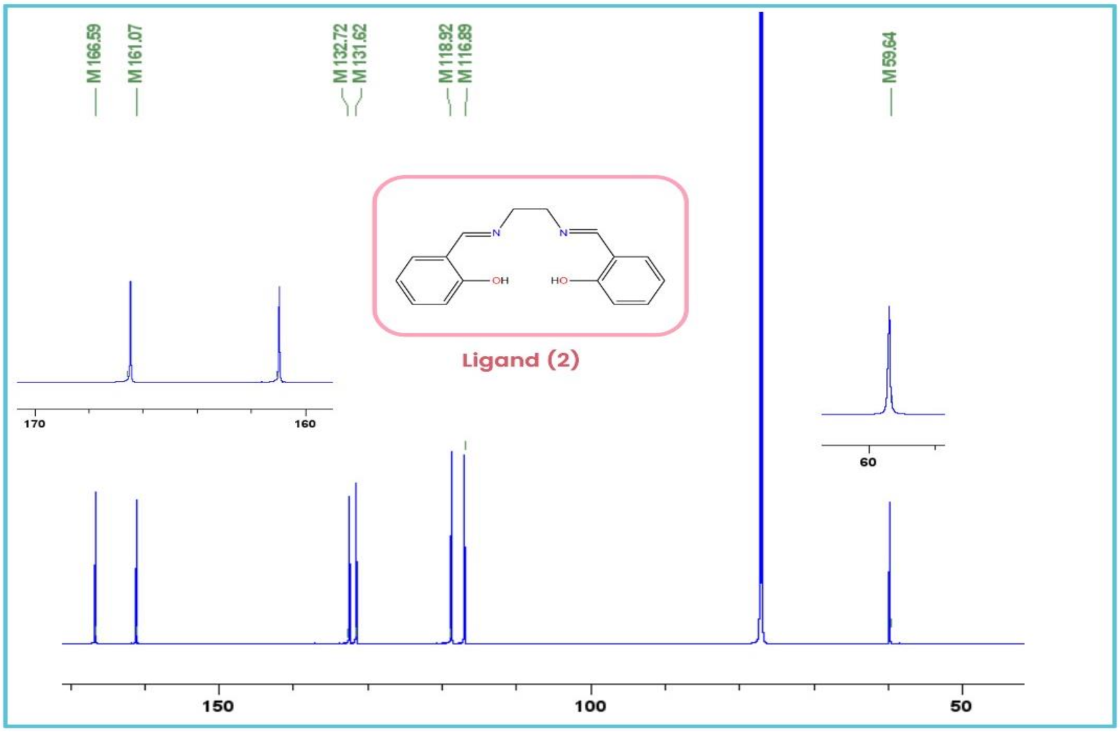

Figure S8. ^{13}C NMR spectrum of ligand (**2**).

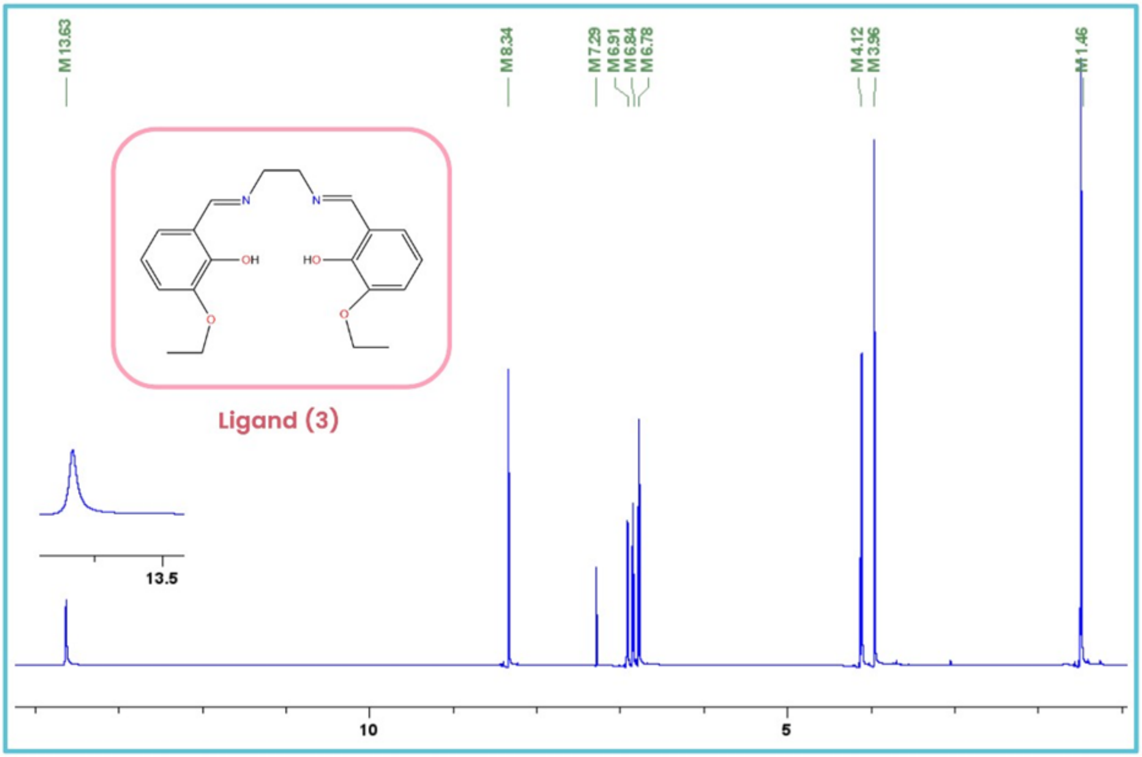

Figure S9. ^{1}H NMR spectrum of ligand (**3**).

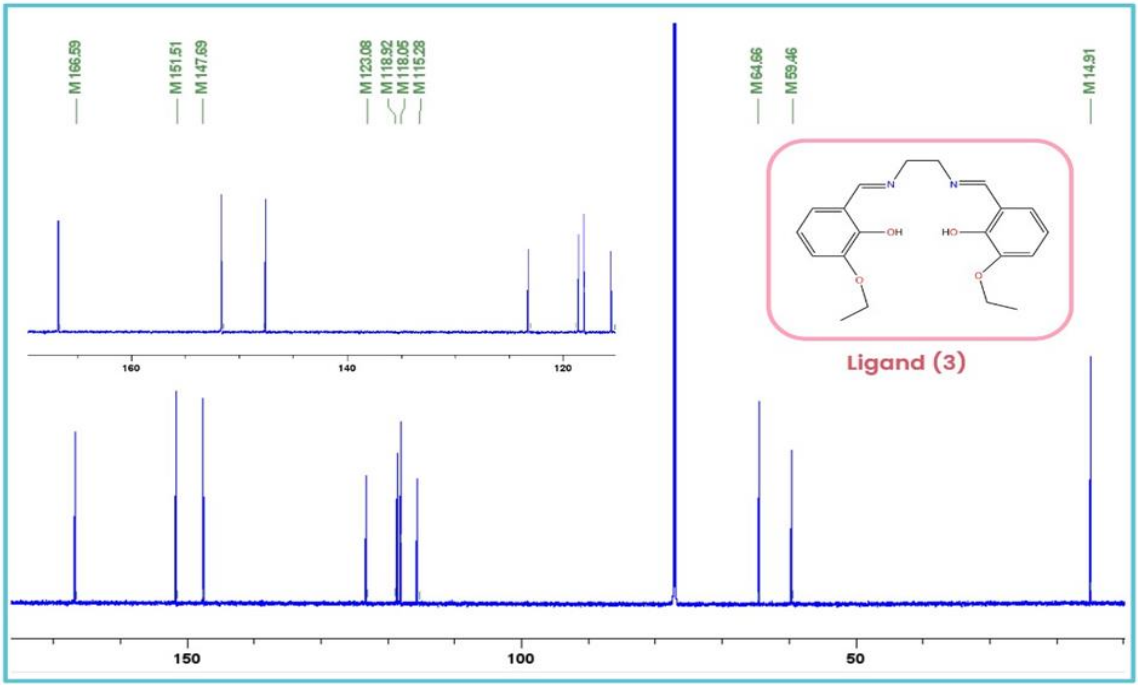

Figure S10. ^{13}C NMR spectrum of ligand (**3**).

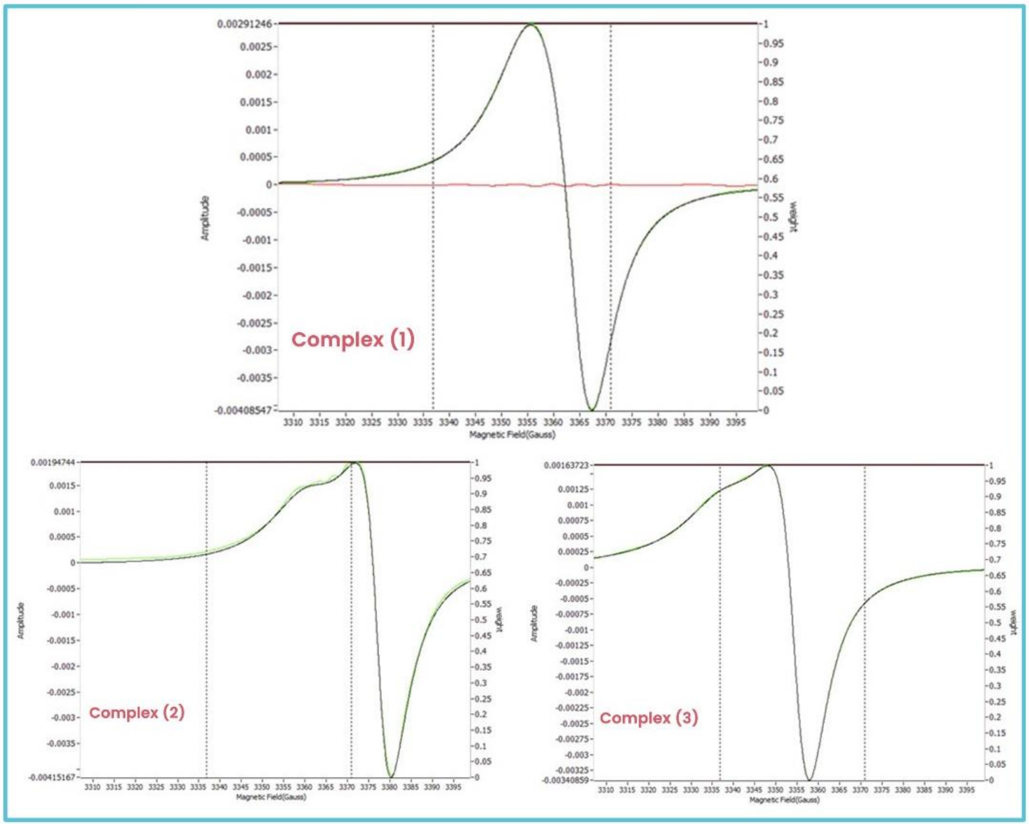

Figure S11. EPR spectra of the copper (II) complexes (**1**-**3**)

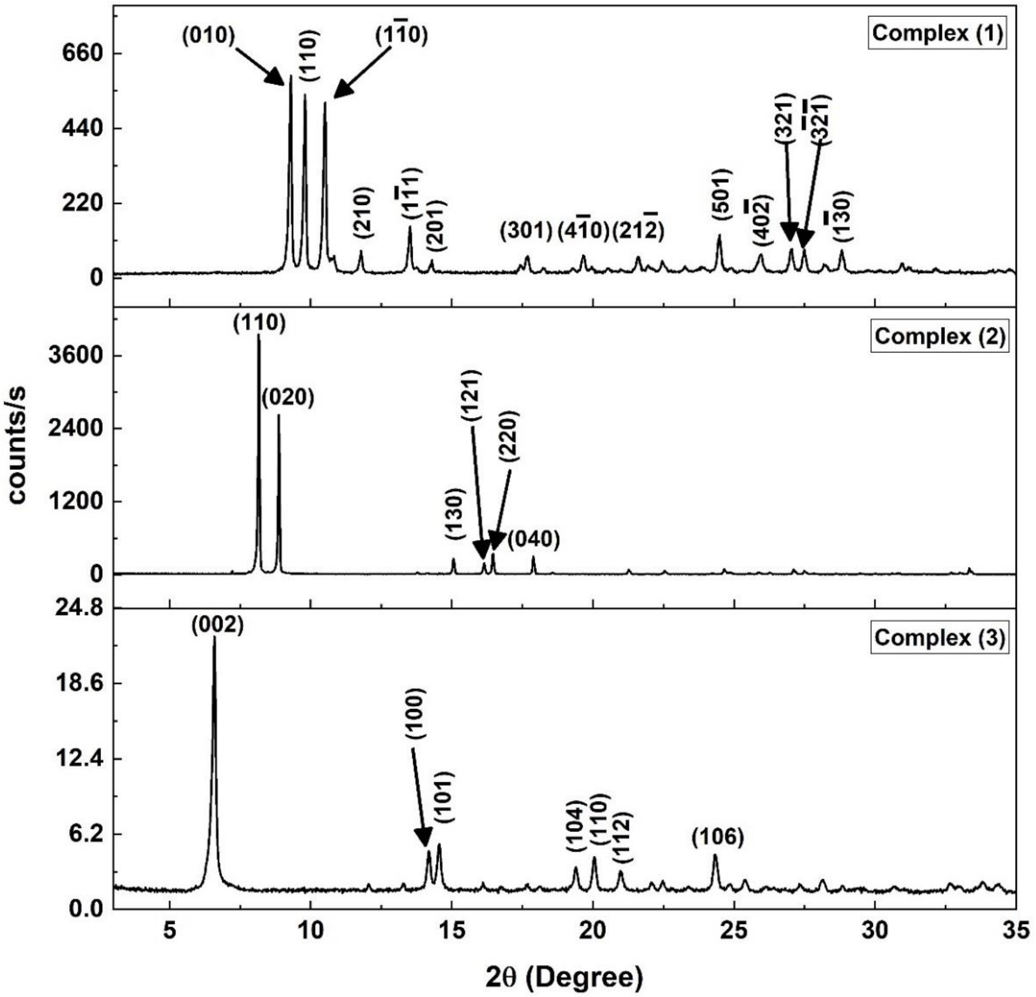

Figure S12. X-ray diffraction patterns of complexes.

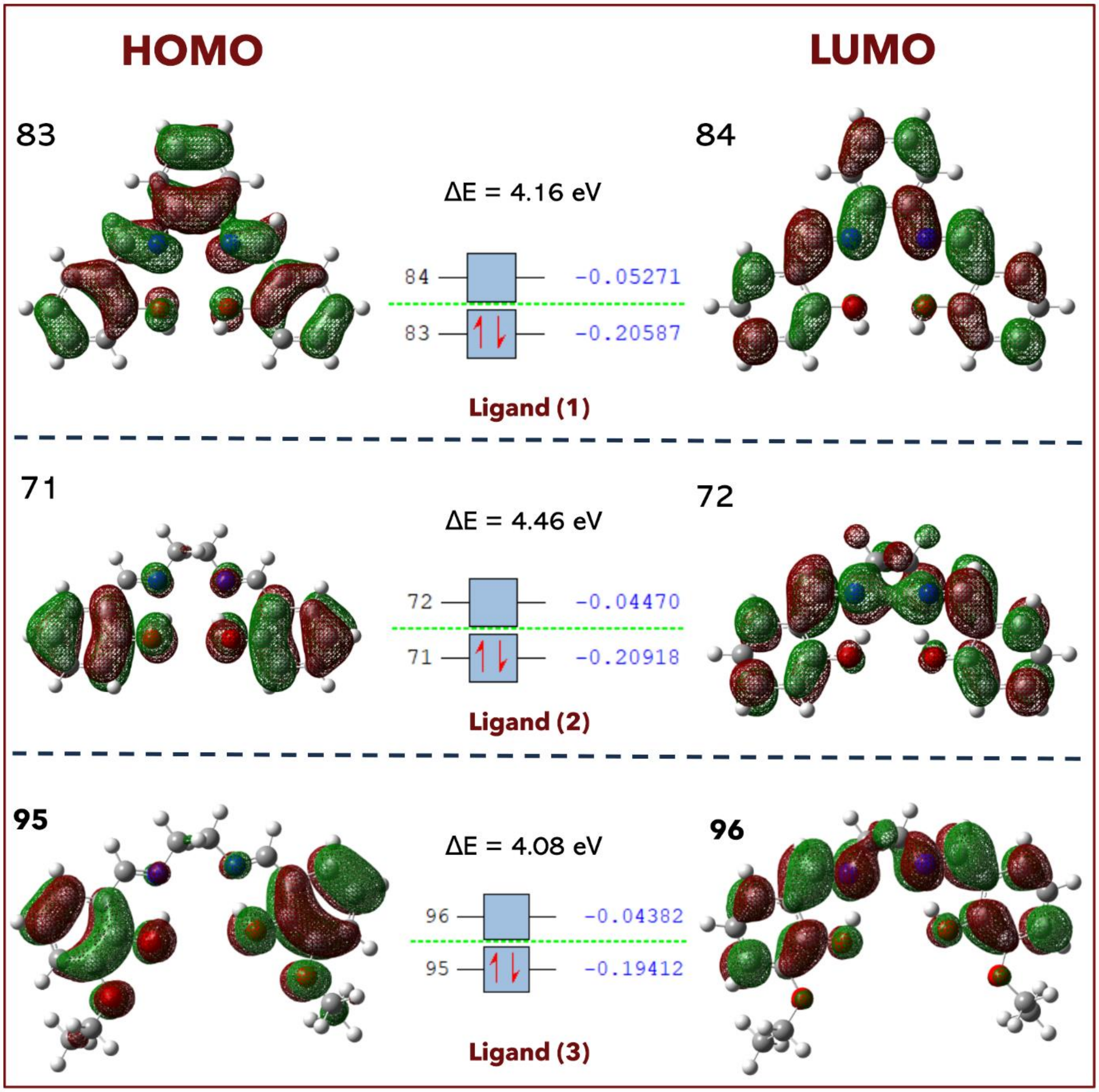

Figure S13. Molecular orbitals (HOMO) and (LUMO) of the ligands (**1**-**3**).

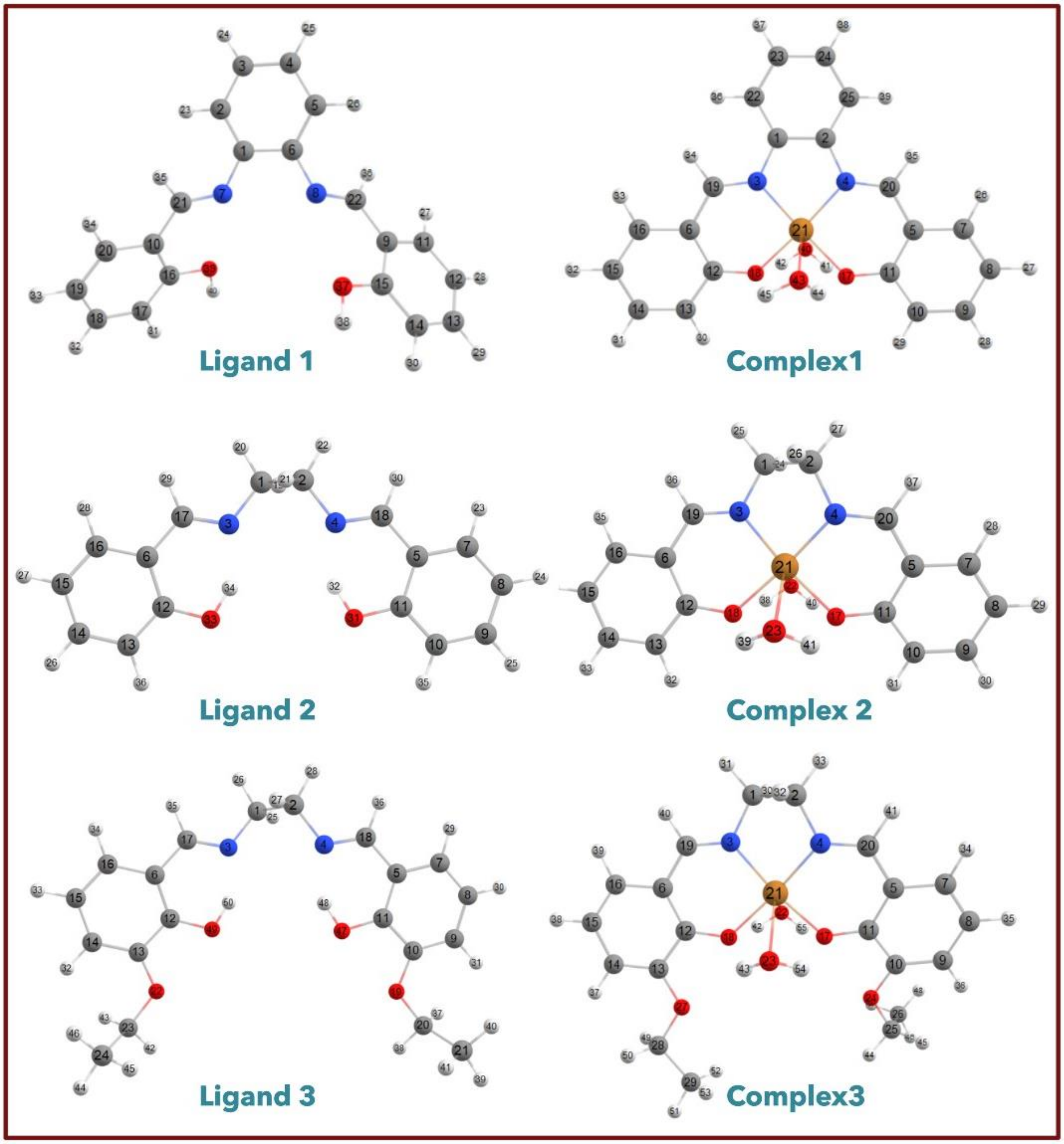

Figure S14. The computed molecular structure of ligands (**1**-**3**) and complexes (**1**-**3**)

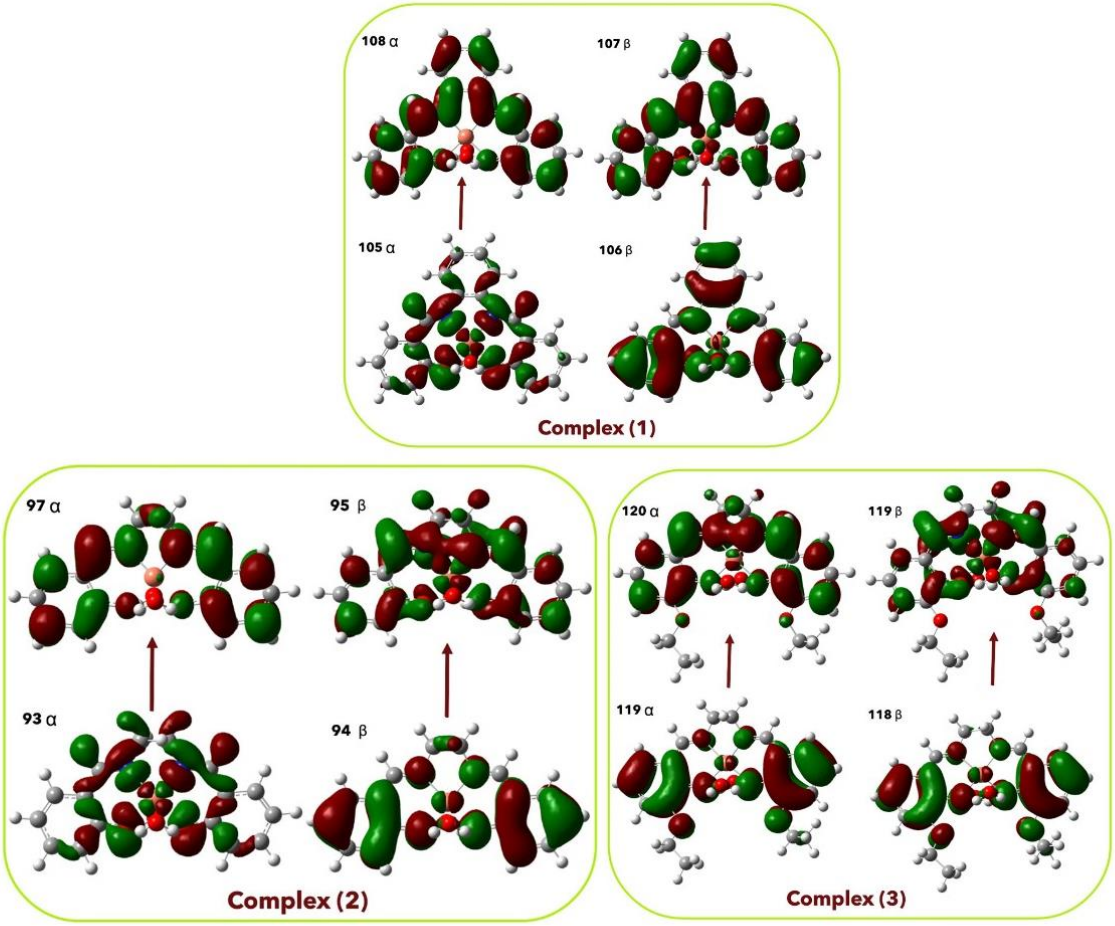

Figure S15. The molecular orbitals which are responsible for the transition of optical absorption.

Tables

Table S1. Experimental data of Cu(II)-ligand complexes (**1**), (**2**) and (**3**) by molar ratio method.

No	Metal Conc. Molar	Ligand Conc. Molar	[M]/([M]+[L])	Complex (1) Absorbance	Complex (2) Absorbance	Complex (3) Absorbance
**1**	0.00001	0	1	0.028	0.028	0.028
**2**	0.00001	0.000005	0.666667	0.064	0.029	0.055
**3**	0.00001	0.00001	0.5	0.097	0.032	0.111
**4**	0.00001	0.000015	0.4	0.117	0.051	0.168
**5**	0.00001	0.00002	0.333333	0.188	0.086	0.216
**6**	0.00001	0.000025	0.285714	0.223	0.108	0.276
**7**	0.00001	0.00003	0.25	0.254	0.138	0.322

Table S2. The selected IR peaks for salen ligands and their Copper complexes

	Comp.	C=N	C-O	Cu-N	Cu-O
**1**	**Ligand 1**	1620	1282	-	-
**2**	**Ligand 2**	1639	1288	-	-
**3**	**Ligand 3**	1637	1260	-	-
**4**	**Complex 1**	1611	1338	501	620
**5**	**Complex 2**	1629	1354	471	618
**6**	**Complex 3**	1623	1312	463	610

Table S3. The energy gap values of the salen ligands and their Copper complexes

Compound	Experimental			Computational			
	Peaks (eV)			Peaks (eV)			
	(1)	(2)	(3)	(1)	(2)	(3)	(4)
**Ligand 1**	5.35	4.03	3.15	5.39	4.14	3.52	2.7
**Ligand 2**	5.32	4.56	3.60	5.24	4.23	3.47	---
**Ligand 3**	5.23	4.34	3.49	5.3	4.17	3.3	---
**Complex 1**	---	---	2.67	---	---	2.61	---
**Complex 2**	---	---	3.10	---	---	3.10	---
**Complex 3**	---	---	3.00	---	---	2.90	---

Table S4. EPR characteristics are determined for Cu(II) complexes.

	Complex **1**	Complex **2**	Complex **3**
$g_{\perp}$	2.004	2.006	2.006
$g_{\parallel}$	2.004	2.025	1.997
g^2_{av}	4.016	4.049	4.012
**G**	1	5.75	-1.25
μ_{eff}	1.73	1.74	1.73

Table S5. Thermal analysis data of salen ligands and their copper(II) complexes.

	Temperature	Wt. % loss Found-Calc.	Assignment
**Ligand 1**	196-327	44.63 - 44.93	$C_6H_{10}N_2O_2$
	327-683	51.79 - 51.26	$C_{13}H_6$
	683-1000	3.58 - 3.81	C
**Complex 1**	306-406	39.58 - 39.67	$C_6H_{16}N_2O_3$
	406-1000	29.23 - 29.51	$C_{10}H_2$
	>1000	31.19 - 30.82	C_4 + CuO (residue)
**Ligand 2**	160 - 322	91.61 - 91.05	$C_{16}H_{16}N_2O_2$
	>322	8.39 - 8.95	C_2
**Complex 2**	304-386	58.33 - 58.56	$C_{10}H_{18}N_2O_3$
	386-800	19.69 - 19.70	C_6
	>800	21.98 - 21.74	CuO (residue)
**Ligand 3**	199-321	61.80 - 61.98	$C_9H_{20}N_2O_4$
	321-640	38.20 - 38.02	$C_{11}H_4$
**Complex 3**	283-376	57.12 - 57.11	$C_{11}H_{19}N_2O_5$
	376-639	25.75 - 25.37	C_9H_7
	639-1000	17.13 - 17.52	CuO (residue)

Table S6. Crystallographic data of the complexes.

**Complex**	**System**	**Space group**	**average crystallite size C_s (nm)**	**Lattice parameters**					
				**a**	**b**	**c**	**A**	**β**	**γ**
				**Å**			**Degree**		
**S1**	Triclinic	P-1	59.5	21.92	9.96	8.52	107.12	98.57	82.36
**2**	Tetragonal	P4/m	48	6.27	6.27	27.12	90	90	90
**3**	Orthorhombic	Pmmm	100	12.8	19.8	7.63	90	90	90

Table S7. Significant bond angles and bond lengths of the ligands and their complexes.

Bond length				Bond angle			
**Ligand 1**		**Complex 1**		**Ligand 1**		**Complex 1**	
O(39)-C(16)	1.430	O(18)-C(12)	1.302	C(9)-C(22)-N(8)	120.000	C(5)-C(20)-N(4)	126.228
O(37)-C(15)	1.430	O(17)-C(11)	1.308	N(7)-C(21)-C(10)	120.000	N(3)-C(19)-C(6)	125.809
C(22)-N(8)	1.294	C(20)-N(4)	1.304	O(39)-C(16)-C(10)	120.000	O(18)-C(12)-C(6)	123.948
C(21)-N(7)	1.294	C(19)-N(3)	1.305	O(37)-C(15)-C(9)	120.000	O(17)-C(11)-C(5)	124.003
N(8)-C(6)	1.470	N(4)-C(2)	1.410	C(21)-C(10)-C(16)	120.000	C(19)-C(6)-C(12)	122.827
N(7)-C(1)	1.470	N(3)-C(1)	1.408	C(22)-C(9)-C(15)	120.000	C(20)-C(5)-C(11)	123.260
				N(8)-C(6)-C(1)	120.000	N(4)-C(2)-C(1)	115.026
				N(7)-C(1)-C(6)	120.000	N(3)-C(1)-C(2)	115.070
**Ligand 2**		**Complex 2**		**Ligand 2**		**Complex 2**	
O(33)-C(12)	1.301	O(18)-C(12)	1.310	N(4)-C(18)-C(5)	120.000	N(4)-C(20)-C(5)	125.622
O(31)-C(11)	1.301	O(17)-C(11)	1.310	C(6)-C(17)-N(3)	120.000	C(6)-C(19)-N(3)	125.620
C(18)-N(4)	1.294	C(20)-N(4)	1.293	O(33)-C(12)-C(6)	120.000	O(18)-C(12)-C(6)	124.180
C(17)-N(3)	1.294	C(19)-N(3)	1.293	O(31)-C(11)-C(5)	120.000	C(5)-C(11)-O(17)	124.183
N(4)-C(2)	1.470	N(4)-C(2)	1.461	C(17)-C(6)-C(12)	120.000	C(19)-C(6)-C(12)	122.514
N(3)-C(1)	1.470	N(3)-C(1)	1.461	C(18)-C(5)-C(11)	120.000	C(11)-C(5)-C(20)	122.517
				C(1)-C(2)-N(4)	109.471	N(4)-C(2)-C(1)	107.378
				C(2)-C(1)-N(3)	109.471	N(3)-C(1)-C(2)	107.383
**Ligand 3**		**Complex 3**		**Ligand 3**		**Complex 3**	
O(49)-C(12)	1.337	O(18)-C(12)	1.305	N(4)-C(18)-C(5)	122.969	N(4)-C(20)-C(5)	125.920
O(47)-C(11)	1.337	O(17)-C(11)	1.305	C(6)-C(17)-N(3)	122.961	C(6)-C(19)-N(3)	125.878
C(18)-N(4)	1.286	C(20)-N(4)	1.294	O(49)-C(12)-C(6)	122.683	O(18)-C(12)-C(6)	124.919
C(17)-N(3)	1.286	C(19)-N(3)	1.294	O(47)-C(11)-C(5)	122.664	C(5)-C(11)-O(17)	124.820
N(4)-C(2)	1.450	N(4)-C(2)	1.461	C(17)-C(6)-C(12)	120.229	C(19)-C(6)-C(12)	121.700
N(3)-C(1)	1.450	N(3)-C(1)	1.460	C(18)-C(5)-C(11)	120.262	C(11)-C(5)-C(20)	121.732
				C(1)-C(2)-N(4)	112.321	N(4)-C(2)-C(1)	107.232
				C(2)-C(1)-N(3)	112.251	N(3)-C(1)-C(2)	107.211

Table S8. NBO Charge distribution of the ligands and their complexes

	N1	N2	O1	O2	Cu^{+2}	O-Water
**Ligand 1**	-0.396	-0.396	-0.674	-0.674		
**Ligand 2**	-0.526	-0.526	-0.692	-0.692		
**Ligand 3**	-0.520	-0.520	-0.680	-0.680		
**Complex 1**	-0.568	-0.569	-0.756	-0.741	1.093	-0.956, -0.957
**Complex 2**	-0.578	-0.578	-0.763	-0.763	1.080	-0.959, -0.960
**Complex 3**	-0.582	-0.582	-0.749	-0.749	1.089	-0.965, -0.965

Table S9. Absorption properties obtained for ADMET prediction

	**Ligand 1**	**Ligand 2**	**Ligand 3**	**Complex 1**	**Complex 2**	**Complex 3**
**Caco-2 Permeability (logPaap)**	-4.47	-4.59	-4.69	-4.64	-4.38	-4.5
Interpretation	None	None	None	None	None	None
**Human Oral Bioavailability 20%**	Bioavailable (0.723)	Bioavailable (0.71)	Bioavailable (0.576)	Non-Bioavailable (0.119)	Non-Bioavailable (0.146)	Non-Bioavailable (0.053)
Interpretation	Medium Confidence	Medium Confidence	Low Confidence	High Confidence	High Confidence	High Confidence
**Human Intestinal Absorption**	Absorbed (0.994)	Absorbed (0.979)	Absorbed (0.95)	Absorbed (0.648)	Absorbed (0.705)	Non-Absorbed (0.387)
Interpretation	High Confidence	High Confidence	High Confidence	Low Confidence	Medium Confidence	Low Confidence
**P-Glycoprotein Inhibitor**	Non-Inhibitor (0.121)	Non-Inhibitor (0.001)	Inhibitor (0.527)	Non-Inhibitor (0.007)	Non-Inhibitor (0.015)	Non-Inhibitor (0.115)
Interpretation	High Confidence	High Confidence	Low Confidence	High Confidence	High Confidence	High Confidence

Table S10. Distribution properties obtained for ADMET prediction

	**Blood-Brain Barrier Penetration**	Interpretation	**Fraction Unbound (Human)**	Interpretation	**Plasma Protein Binding (%)**	Interpretation
**Ligand 1**	Penetrable (0.553)	Low Confidence	1.19	None	92.24	Poor Value >90%
**Ligand 2**	Non-Penetrable (0.447)	Low Confidence	0.89	None	65.9	Therapeutic Index <90%
**Ligand 3**	Non-Penetrable (0.476)	Low Confidence	1.02	None	72.82	Therapeutic Index <90%
**Complex 1**	Penetrable (1.0)	High Confidence	2.12	None	37.58	Therapeutic Index <90%
**Complex 2**	Penetrable (1.0)	High Confidence	1.38	None	28.76	Therapeutic Index <90%
**Complex 3**	Penetrable (0.995)	High Confidence	1.42	None	30.6	Therapeutic Index <90%

Table S11. Metabolism properties obtained for ADMET prediction

	**CYP3A4 Inhibitor**	**Interpretation**	**CYP2D6 Substrate**	**Interpretation**
**Ligand 1**	Non-Inhibitor (0.002)	High Confidence	Substrate (0.52)	Low Confidence
**Ligand 2**	Non-Inhibitor (0.0)	High Confidence	Substrate (0.554)	Low Confidence
**Ligand 3**	Non-Inhibitor (0.051)	High Confidence	Substrate (0.51)	Low Confidence
**Complex 1**	Non-Inhibitor (0.0)	High Confidence	Non-Substrate (0.044)	High Confidence
**Complex 2**	Non-Inhibitor (0.0)	High Confidence	Non-Substrate (0.085)	High Confidence
**Complex 3**	Non-Inhibitor (0.01)	High Confidence	Non-Substrate (0.077)	High Confidence

Table S12. Excretion properties obtained for ADMET prediction

	**Clearance**	Interpretation	**Half-Life**	Interpretation
**Ligand 1**	5.64	None	<3h (0.085)	High Confidence
**Ligand 2**	5.5	None	<3h (0.198)	Medium Confidence
**Ligand 3**	5.7	None	<3h (0.207)	Medium Confidence
**Complex 1**	8.28	None	<3h (0.109)	High Confidence
**Complex 2**	8.72	None	<3h (0.155)	High Confidence
**Complex 3**	9.09	None	<3h (0.1)	High Confidence

References:

[1] E. M. M. Ali, A. A. Elashkar, H. Y. El-Kassas, E. I. Salim, *Int J Biol Macromol* **2018**, *120*, 1170–1180.

[2] S. P. Langdon, *Basic Principles of Cancer Cell Culture." Cancer Cell Culture: Methods and Protocols.*, **2004**.

[3] Debye P and Scherrer P, *Phys. Zeitschrift* **1916**, *17*.
